# Epigenetic and RNA-mediated regulation of plant VOC biosynthesis and signalling

**DOI:** 10.3389/fpls.2026.1945830

**Published:** 2026-10-20

**Authors:** Aleksandra Boba, Anna Domańska, Anna Kulma, Kamila Nowosad, Henryk Bujak, Kamil Kostyn

**Affiliations:** 1 Faculty of Biotechnology, University of Wroclaw, Wroclaw, Poland; 2 Department of Genetics, Plant Breeding and Seed Production, Faculty of Life Sciences and Technology, Wroclaw University of Environmental and Life Sciences, Wroclaw, Poland; 3 Research Centre for Cultivar Testing, Słupia Wielka, Poland

**Keywords:** defence priming, DNA methylation, epitranscriptomics, histone modifications, m^6^A, non-coding RNA, VOC signalling, volatile organic compounds (VOCs)

## Abstract

Plants use volatile organic compounds (VOCs) as airborne signals in development, defence, and ecological interactions. Epigenetic mechanisms can influence VOC biosynthesis and, in some experimental systems, plant responses to volatile cues. This review examines chromatin-level mechanisms, particularly DNA methylation and histone modifications, in VOC biosynthesis and responses to volatile cues, together with distinct RNA-level regulation by non-coding RNAs and m^6^A-dependent epitranscriptomic mechanisms. VOC perception can activate rapid signalling events, including Ca^2+^ influx and reactive oxygen species production; however, direct mechanistic links between these early responses and subsequent chromatin changes remain unresolved. Changes in promoter DNA methylation and histone acetylation are associated with, and in some systems functionally contribute to, regulation of VOC biosynthesis or defence responses, whereas ncRNA- and m^6^A-dependent mechanisms provide distinct RNA-level regulatory layers. Across diverse plant systems, chromatin states can influence VOC biosynthesis and emission, whereas in a smaller set of exposure studies, volatile cues are accompanied by changes in DNA methylation or histone modifications. Persistent or primed responses have been reported in several systems, but persistence of a phenotype or chromatin mark alone does not establish stress memory, and causal epigenetic involvement in VOC-induced memory remains unresolved. Important knowledge gaps concern the mechanisms linking volatile perception with chromatin regulation, the integration of complex VOC blends, and the relevance of these processes under field conditions. Emerging approaches, including locus-specific CRISPR/dCas-based epigenome editing and temporally resolved multi-omics, should help distinguish causal chromatin mechanisms from downstream transcriptional responses. Current evidence therefore supports distinct chromatin- and RNA-level contributions to VOC biology but does not yet establish a general mechanistic pathway from volatile perception to epigenetically encoded stress memory.

## Highlights

- Chromatin-level epigenetic mechanisms contribute to plant VOC biosynthesis in selected systems and are associated with persistent or primed responses to volatile cues, but their causal contribution to VOC-induced stress memory remains unresolved.

## Introduction

Plants use volatile organic compounds (VOCs) as airborne signalling molecules to communicate with their surrounding environment. Unlike communication based on direct contact, such as common mycorrhizal networks, VOC signalling enables rapid information transfer without direct physical contact between the emitter and receiver (Šimpraga et al., 2016; Sharifi and Ryu, 2021). Plant VOCs perform diverse ecological functions, including attracting pollinators, mediating direct and indirect defence against herbivores, and conveying information between neighbouring plants (Lo et al., 2024; Rashmi et al., 2026). Their emission is influenced by environmental factors such as temperature, humidity and light, as well as by plant age, developmental stage and tissue identity. Despite this ecological understanding, the molecular mechanisms linking VOC signalling to changes in gene regulation remain incompletely understood. Recent studies indicate that VOC production and emission are influenced by epigenetic mechanisms, particularly DNA methylation and histone modifications, as well as by ncRNA-mediated regulatory processes acting predominantly at the post-transcriptional level (Xie et al., 2024; Catoni et al., 2025). Changes in chromatin accessibility are discussed where they have been directly measured at VOC-related loci; however, direct evidence for the involvement of ATP-dependent chromatin-remodelling complexes in VOC regulation remains limited. VOC-mediated defence and priming have clear ecological and physiological effects, but the mechanisms responsible for responses that persist after volatile exposure remain poorly understood. Few studies test whether chromatin changes induced by a defined VOC are required for altered responses to subsequent challenge. Epigenetic regulation should therefore be considered a candidate mechanism for persistent or primed VOC responses rather than an established mechanism of VOC-induced stress memory. It also remains unclear which VOCs induce specific chromatin changes, how long these changes persist, whether they occur locally or systemically, and whether any VOC-associated epigenetic states are transmitted to subsequent generations (Picazo-Aragonés et al., 2020; Dobránszki et al., 2025).

In this review, epigenetic regulation refers to changes in DNA methylation or chromatin state that alter gene regulation without changing the underlying DNA sequence (Lämke and Bäurle, 2017). Non-coding RNAs are classified according to their mechanism of action. Chromatin-associated ncRNA functions, including RNA-directed DNA methylation (RdDM), are considered epigenetic when they directly modify DNA methylation or chromatin state (Erdmann and Picard, 2020; Wierzbicki et al., 2021). By contrast, miRNA-mediated transcript cleavage and translational repression are treated as post-transcriptional regulation rather than epigenetic regulation per se (Yu et al., 2017). Similarly, lncRNA effects on transcript processing, stability or translation are classified as RNA-level regulation unless a direct chromatin-associated mechanism is demonstrated (Wierzbicki et al., 2021). m^6^A-dependent regulation is considered epitranscriptomic RNA-level regulation and is therefore discussed separately from chromatin-level epigenetic mechanisms.

Throughout this review, we distinguish association, functional involvement and locus-specific epigenetic causality. Association describes cases in which a chromatin change coincides with a transcriptional, metabolic or physiological response without evidence that it drives that response. Functional involvement refers to studies in which genetic or pharmacological perturbation of a relevant regulatory component or machinery alters the response. Locus-specific epigenetic causality requires targeted manipulation of a defined chromatin or DNA-methylation state at a defined locus, followed by demonstration of the corresponding functional outcome. We use priming when a prior treatment alters the response to a subsequent challenge, for example by producing a faster or stronger response. This is distinct from a persistent response, in which molecular, physiological or phenotypic effects of the initial treatment remain detectable after the treatment has ended. Stress memory refers here to an altered response retained after a recovery period and revealed by a subsequent challenge. Persistence of a DNA-methylation state, histone modification, transcript profile or phenotype after cue removal is therefore not sufficient, by itself, to demonstrate stress memory. Because the term “short-term memory” is used inconsistently, we do not use it for persistence alone. Transgenerational inheritance refers to effects detected in descendants that were not directly exposed to the initiating treatment. Current evidence does not establish VOC-specific transgenerational inheritance (Lämke and Bäurle, 2017; Dobránszki et al., 2025).

Accordingly, this review considers two complementary aspects of VOC regulation. We first summarise the major VOC biosynthetic pathways and examine how DNA methylation, histone modifications and distinct RNA-level mechanisms, including ncRNA- and m^6^A-dependent regulation, influence VOC biosynthesis and emission across developmental and stress-related contexts. We then focus on responses to volatile exposure, from perception and early cellular signalling to the available evidence for subsequent chromatin- and RNA-level changes in receiver tissues. Finally, we evaluate persistent and primed responses following volatile exposure, assess the extent to which current data support stress memory or transgenerational inheritance, and identify the major mechanistic and ecological gaps that remain.

This narrative review adopts a concept-driven rather than systematic approach to the literature. The literature considered spans foundational studies from the early 2000s through work published in 2026, with particular emphasis on recent primary experimental studies. Relevant publications were identified primarily through Google Scholar and PubMed using combinations of terms including “plant VOCs”, “volatile signalling”, “DNA methylation”, “histone modification”, “histone acetylation”, “chromatin”, “miRNA”, “lncRNA”, “ncRNA”, “m^6^A”, “priming” and “stress memory”. Additional targeted searches were performed for individual volatile compounds, genes, regulatory components and plant species where required. Because studies directly connecting volatile exposure with chromatin regulation remain limited and span heterogeneous experimental designs, formal systematic-review inclusion criteria were not applied. Studies directly linking VOC biology with chromatin- or RNA-level regulation formed the core of the review. Selected studies of early signalling and ecological interactions were included to provide mechanistic and field-relevant context. Recent primary studies were prioritised, whereas earlier experimental studies and selected reviews were used where necessary to establish conceptual or broader background context. The aim was not exhaustive coverage, but a critical integration of the available evidence across molecular, physiological and ecological levels.

## Plant volatile organic compounds

The volatility of plant VOCs is primarily determined by their relatively high vapour pressure and low boiling point, properties that allow these compounds to enter the gas phase under physiological and environmental conditions (Dudareva et al., 2006; Liu et al., 2023). Plants release VOCs from both reproductive organs, particularly flowers, and vegetative tissues such as leaves, fruits and roots (Dudareva et al., 2006; Maffei, 2010). In many flowers, VOCs are synthesised in epidermal or other specialised tissues and subsequently diffuse into the surrounding air. In vegetative organs, VOC production and storage can also be spatially compartmentalised (Maffei, 2010). In conifers, for example, terpenoid-containing resin can be stored in specialised structures such as resin ducts. Scots pine (*Pinus sylvestris*) emits VOCs from both *de novo* synthesised and stored pools, with emissions from stored pools associated with resin-storage structures (Maffei, 2010; Yu et al., 2022). VOCs may also be retained in non-volatile conjugated forms. In tea (*Camellia sinensis*) leaves, monoterpene, aromatic and aliphatic alcohols are accumulated predominantly as water-soluble glycosides, particularly β-primeverosides, which can be enzymatically hydrolysed to release the corresponding volatile aglycones (Ohgami et al., 2015). VOC biosynthesis and emission also depend on precursor availability and energy supplied by primary metabolism (Dudareva et al., 2006).

VOC emission patterns depend on the physiological and developmental state of the plant (Liu et al., 2023). In vegetative tissues, VOC emission is closely linked to defence, whereas floral VOCs frequently function in pollinator attraction (Dudareva et al., 2006; Maffei, 2010). Stress can induce *de novo* VOC production and alter the composition and abundance of the emitted blend (Maffei, 2010). Different volatile cues can activate distinct, although partly overlapping, defence and signalling pathways (Erb, 2018). Temporal regulation adds another level of control. Light strongly influences the diurnal emission of herbivore-induced VOCs in wild tobacco (He et al., 2021), whereas in petunia floral VOC biosynthesis and emission are coordinated with the day–night cycle through transcriptional regulation and dynamic histone acetylation (Patrick et al., 2021; Patrick et al., 2024).

### Major biosynthetic pathways of plant VOCs

A survey of 991 flowering-plant species and several gymnosperms from 90 seed-plant families identified more than 1,700 floral volatile compounds (Knudsen et al., 2006). Many additional floral volatiles have been identified since, including compounds of relatively low volatility that were poorly captured by earlier headspace-based surveys (Dötterl and Gershenzon, 2023). Vegetative organs, including leaves, stems and roots, emit an additional and highly context-dependent diversity of volatile compounds, whose profiles vary with tissue, genotype, developmental state and environmental conditions (Escobar-Bravo et al., 2023). Consequently, the total size of the plant volatilome cannot presently be defined precisely. Plant VOCs can be grouped into several broad biosynthetic classes, including phenylpropanoids and benzenoids, terpenoids, carotenoid-derived apocarotenoids, fatty acid-derived VOCs, amino acid-derived volatiles, and low-molecular-weight oxygenated VOCs. These categories are simplified and not mutually exclusive, and individual compounds may differ in biosynthetic origin and biological function depending on the plant system. Representative pathways, compounds and functions are summarised in **Figure 1** and **Table 1** (Pichersky et al., 2006; Dötterl and Gershenzon, 2023; Escobar-Bravo et al., 2023).

**Figure 1:**
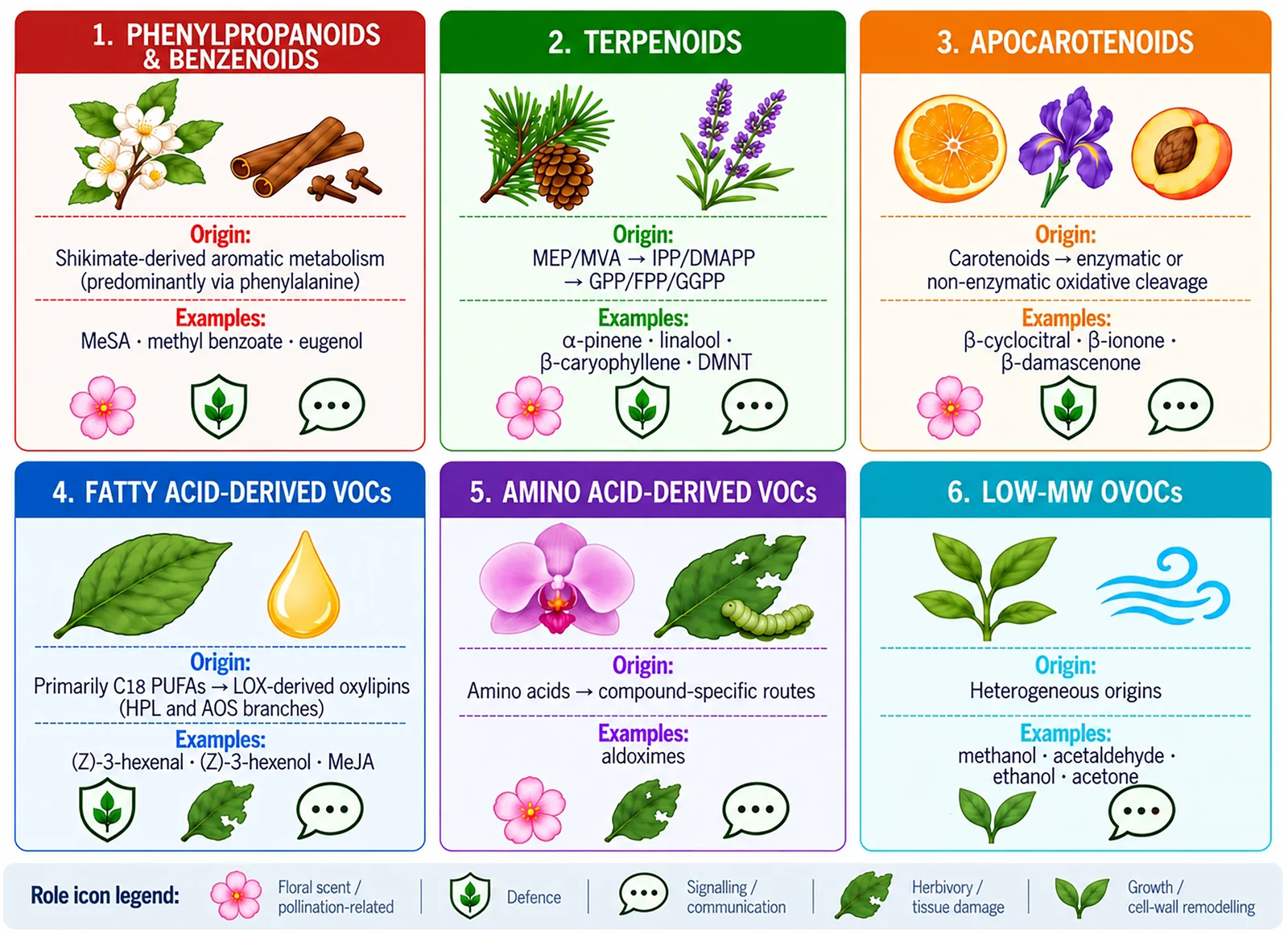
Major biosynthetic classes of plant volatile organic compounds (VOCs). Plant VOCs comprise phenylpropanoids and benzenoids, terpenoids, apocarotenoids, fatty acid-derived VOCs, amino acid-derived VOCs, and low-molecular-weight oxygenated VOCs (OVOCs). Representative biosynthetic origins, compounds, and major biological or ecological roles are shown. The classification is simplified and illustrative rather than exhaustive; expanded coverage is provided in **Table 1**.

**Table 1:** Major biosynthetic classes of plant volatile organic compounds (VOCs), their biosynthetic origins, representative compounds, biological functions and key references.

VOC class	Major biosynthetic origin and key route	Representative compounds	Representative biological/ecological roles	Key refs.
Phenylpropanoids and benzenoids	Derived predominantly from phenylalanine supplied by the shikimate pathway. Branch-specific side-chain modification, shortening, methylation, reduction or acylation generates diverse volatile benzenoids and phenylpropenes.	Methyl salicylate (MeSA), methyl benzoate, benzyl acetate, eugenol, isoeugenol, phenylacetaldehyde	Major contributors to floral and fruit scent; pollinator attraction; direct defence against herbivores or microorganisms; airborne defence signalling and plant–plant communication for selected compounds such as MeSA.	(Pichersky et al., 2006; Cheng et al., 2023; Dötterl and Gershenzon, 2023; Escobar-Bravo et al., 2023)
Terpenoids (isoprenoids)	Formed from IPP and DMAPP generated through the plastidial MEP and cytosolic MVA pathways. GPP, FPP and GGPP provide substrates for terpene synthases; homoterpenes arise through further oxidative processing of terpene precursors.	Isoprene, α-pinene, limonene, linalool, β-caryophyllene, (E)-nerolidol, DMNT, TMTT	Floral and vegetative scent; pollinator attraction; direct and indirect defence; attraction of natural enemies of herbivores; plant–plant signalling; abiotic-stress protection for selected volatile isoprenoids.	(Pichersky et al., 2006; Maffei, 2010; Bruce and Pickett, 2011; Rosenkranz et al., 2021)
Apocarotenoids	Produced by carotenoid cleavage, either enzymatically through carotenoid cleavage dioxygenases (CCDs) or non-enzymatically through oxidative cleavage by ROS, including ¹O_2_.	β-Cyclocitral, β-ionone, β-damascenone, safranal	Floral and fruit aroma; stress-associated and plastid-to-nucleus signalling; regulation of acclimation responses for selected compounds.	(Ramel et al., 2012; Carnà et al., 2025)
Fatty acid-derived VOCs: green leaf volatiles and volatile oxylipins	Primarily derived from C18 polyunsaturated fatty acids, including α-linolenic and linoleic acids. The LOX–HPL branch generates C6 GLVs, whereas the AOS/AOC/OPR branch generates jasmonates; JA can be methylated to MeJA.	(Z)-3-Hexenal, (E)-2-hexenal, (Z)-3-hexenol, (Z)-3-hexenyl acetate, (Z)-3-hexenyl butyrate, methyl jasmonate (MeJA)	Rapid responses to tissue damage and herbivory; direct and indirect defence; within-plant and plant–plant signalling; defence priming; interaction with jasmonate-dependent defence networks.	(Jiang et al., 2017; Matsui and Engelberth, 2022; Payá et al., 2023)
Amino acid-derived VOCs	Derived from amino acids and related intermediates through compound-specific biosynthetic routes.	Indole, phenylacetaldehyde, phenylacetaldoxime, 2-methylbutyraldoxime, 3-methylbutyraldoxime	Components of floral scent in selected taxa; herbivore-induced volatiles; cues in insect–parasitoid interactions. Some aldoximes also serve as intermediates in the biosynthesis of non-volatile defence metabolites.	(Pichersky et al., 2006; Dötterl and Gershenzon, 2023; Escobar-Bravo et al., 2023)
Low-molecular-weight oxygenated VOCs (OVOCs; heterogeneous origins)	A physicochemical rather than a single biosynthetic class. Methanol is associated primarily with pectin demethylesterification and cell-wall processes; acetaldehyde and ethanol can arise from fermentative metabolism, whereas acetone has multiple reported plant sources.	Methanol, acetaldehyde, ethanol, acetone	Associated with growth, cell-wall remodelling, wounding and primary-metabolic or stress responses. Signalling roles have been reported for selected compounds but are less consistently established across this heterogeneous group.	(Maffei, 2010; Erb, 2018; Liu et al., 2023)

VOC classes are defined here primarily according to biosynthetic origin and are not mutually exclusive. Apocarotenoids are carotenoid-derived isoprenoids, whereas low-molecular-weight OVOCs comprise compounds of heterogeneous biosynthetic origin. Representative compounds and functions are illustrative rather than exhaustive, and individual VOCs may perform different functions depending on species, tissue, developmental stage and environmental context.

Phenylpropanoid and benzenoid VOCs are synthesised predominantly from phenylalanine supplied by the shikimate pathway and constitute major components of floral scent in many angiosperms. Members of this group contribute to functions including pollinator attraction and plant defence (Pichersky et al., 2006; Dötterl and Gershenzon, 2023). Methyl salicylate (MeSA) can also act as an airborne defence signal. In citrus, controlled airborne MeSA exposure induced defence-related gene expression in receiver plants (Cheng et al., 2023).

Terpenoid VOCs are derived from the C5 precursors isopentenyl diphosphate (IPP) and dimethylallyl diphosphate (DMAPP), generated mainly through the cytosolic mevalonate (MVA) and plastidial methylerythritol phosphate (MEP) pathways. These precursors give rise to mono-, sesqui- and diterpenoid pathways with diverse ecological functions (Pichersky et al., 2006; Liu et al., 2023). Monoterpenes such as α-pinene and limonene are common components of leaf emissions, whereas sesquiterpenes such as β-caryophyllene can contribute to floral scent and indirect defence (Rosenkranz et al., 2021). Homoterpenes such as DMNT and TMTT are frequently emitted following herbivore attack and can contribute to herbivore-induced volatile blends involved in indirect defence and tritrophic interactions (Bruce and Pickett, 2011). Apocarotenoids are generated by carotenoid cleavage through enzymatic reactions catalysed by carotenoid cleavage dioxygenases or through non-enzymatic oxidative processes. Volatile apocarotenoids include β-ionone, β-damascenone and β-cyclocitral. Depending on the compound and biological context, they contribute to floral and fruit aroma or participate in stress-associated signalling; β-cyclocitral is a well-characterised carotenoid oxidation product involved in plastid-to-nucleus stress signalling (Ramel et al., 2012; Carnà et al., 2025).

Fatty acid-derived VOCs arise primarily from C18 polyunsaturated fatty acids through oxylipin-related pathways. The lipoxygenase–hydroperoxide lyase (LOX–HPL) branch generates C6 green leaf volatiles (GLVs), including (Z)-3-hexenal and (Z)-3-hexenol, which can be rapidly released following tissue damage. GLVs function as damage-associated cues and can modulate local and systemic defence responses in both emitting and neighbouring plants (Matsui and Engelberth, 2022). A separate oxylipin branch converts α-linolenic acid through the AOS/AOC/OPR pathway to jasmonic acid (JA), which can subsequently be methylated to the volatile derivative methyl jasmonate (MeJA). JA and MeJA participate broadly in the regulation of plant defence responses (Jiang et al., 2017).

VOC biosynthetic pathways are distinct rather than components of a single integrated route, but their outputs can be coordinated through precursor availability, competition for shared metabolic intermediates, subcellular compartmentation, and regulation of pathway-specific gene expression. This coordination is particularly relevant when primary metabolism supplies common precursors to several branches of specialised metabolism (Dudareva et al., 2006). Experimental studies in petunia further indicate that chromatin-level regulation can coordinate precursor-supplying metabolism with downstream phenylpropanoid/benzenoid VOC biosynthesis (Patrick et al., 2021), a mechanism discussed in detail below.

## Epigenetic and post-transcriptional regulation of VOC biosynthetic bottlenecks

VOC biosynthesis is regulated through transcriptional, chromatin- and RNA-level mechanisms. Recent work has also identified promoter architecture and higher-order chromatin organisation as regulatory features that warrant further investigation in VOC biosynthetic pathways (Lopez-Gordillo et al., 2026). DNA methylation and histone modifications can alter chromatin state and accessibility, whereas most ncRNA-mediated mechanisms described in VOC biosynthesis act at the RNA level. m^6^A-dependent epitranscriptomic regulation provides an additional RNA-level mechanism by modulating transcript stability and translation. These regulatory layers are mechanistically distinct but can intersect, most clearly through RNA-directed DNA methylation, in which small RNAs guide DNA methylation at specific genomic regions (Erdmann and Picard, 2020; Wierzbicki et al., 2021). Effects on VOC-related genes vary by locus, genomic context and biological system (**Figure 2**).

**Figure 2:**
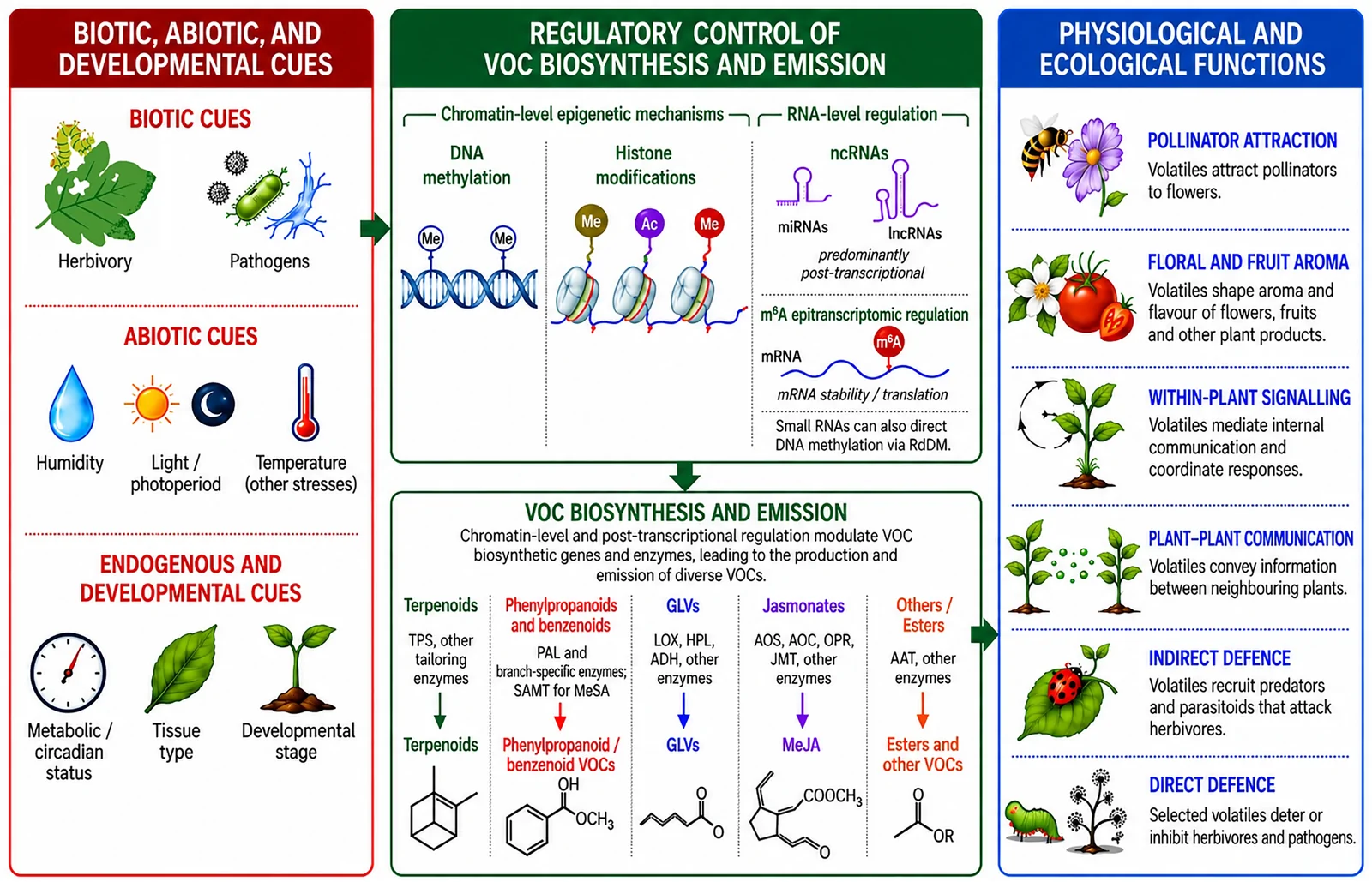
Regulatory control of plant VOC biosynthesis and emission, with downstream biological functions. Biotic, abiotic, endogenous, and developmental cues can influence chromatin-level mechanisms, including DNA methylation and histone modifications, as well as RNA-level regulation by ncRNAs and m^6^A-dependent epitranscriptomic mechanisms. These regulatory layers modulate VOC biosynthetic genes and consequently affect the composition and emission of volatile blends. Plant VOCs contribute to floral and fruit aroma, pollinator attraction, within-plant and plant-to-plant signalling, and direct and indirect defence.

### DNA methylation

DNA methylation in flowering plants occurs in CG, CHG and CHH sequence contexts, and its regulatory consequences depend strongly on genomic location. Promoter methylation, particularly when it affects regulatory regions, is often associated with reduced transcription, whereas CG methylation within gene bodies can occur in actively expressed genes and is not uniformly repressive (Muyle et al., 2022; Qiao et al., 2025). By contrast, CHG and CHH methylation is particularly enriched at transposable elements and other repetitive sequences, where it contributes to transcriptional silencing and genome stability; CHH methylation can also be established through RNA-directed DNA methylation (Erdmann and Picard, 2020; Qiao et al., 2025). These context-dependent effects are important when interpreting methylation changes at VOC-related loci, because the transcriptional consequence of altered methylation depends on both sequence context and genomic position.

#### Developmental and cultivar-specific control of floral VOCs

Across developmental stages and cultivars, DNA-methylation differences at VOC-related loci are associated with changes in gene expression and aroma profiles, but the causal contribution of methylation remains unresolved. In *Prunus mume*, differential DNA methylation during flowering was detected at 22 candidate genes associated with phenylpropanoid/benzenoid floral-scent biosynthesis. These included PmCFAT-family loci (coniferyl alcohol acetyltransferases) associated with eugenol biosynthesis and the functionally characterised benzyl alcohol acetyltransferases PmBEAT36 and PmBEAT37 involved in benzyl acetate formation. Additional candidate genes from the phenylpropanoid/benzenoid pathway included PAL (phenylalanine ammonia-lyase), PAAS (phenylacetaldehyde synthase), BAR (benzaldehyde reductase) and CNL (cinnamate:CoA ligase) family members (Bao et al., 2019; Zhang et al., 2019; Yuan et al., 2021). PmBEAT36 showed promoter CG hypomethylation together with increased expression, whereas PmBEAT37 expression increased despite CHH hypermethylation of its promoter and CG hypomethylation within its gene body (Yuan et al., 2021). Independent functional assays showed that altered PmBEAT36 and PmBEAT37 expression affected benzyl acetate production in petal cells (Bao et al., 2019). Thus, the combined evidence links locus-specific methylation patterns with transcriptional differences but does not establish DNA methylation as the causal regulator of PmBEAT expression. Cultivar-specific aroma profiles have also been associated with differences in DNA methylation and chromatin accessibility at terpene synthase loci. In *Osmanthus fragrans*, TPS2, a key terpene synthase gene in the linalool biosynthetic pathway, showed lower promoter methylation and greater chromatin accessibility in ‘Chenghong Dangui’ than in ‘Zaohuang’, coinciding with higher TPS2 expression and greater accumulation of linalool-related volatiles (Han et al., 2022). In single-petal jasmine ‘Bijian’ (*Jasminum sambac*), integrated metabolomic and transcriptomic profiling across five flower-opening stages identified 15 key aroma volatiles, including several terpenoids. Whole-genome bisulfite sequencing comparing the bud and full-flowering stages showed an overall increase in 5-methylcytosine levels. Higher CHH methylation within the gene bodies of selected terpene synthase genes coincided with increased transcript abundance and was confirmed by bisulfite sequencing PCR. Because the developmental stages were sampled at successive time points during the same night, developmental and diurnal effects cannot be fully distinguished. These findings therefore support an association between DNA-methylation dynamics and stage-dependent floral scent formation, but do not establish DNA methylation as the causal driver of these changes (Zhang et al., 2026).

#### Ripening-associated methylation dynamics and RdDM

In fruit-bearing species, dynamic DNA methylation patterns are associated with, and in some systems functionally contribute to, the regulation of VOC biosynthesis during ripening. In peach, two transcriptional modules link ripening with linalool biosynthesis. Wei et al. (2022) showed that the AP2/ERF-family transcription factor PpERF61 directly activates PpTPS1 and PpTPS3. During ripening, increased PpERF61 expression was associated with reduced methylation of the PpERF61 promoter. In a separate study, Wei et al. (2021) showed that the bHLH-family transcription factor PpbHLH1 directly activates PpTPS3, whilst reduced PpTPS3 promoter methylation was associated with increased PpTPS3 expression and linalool accumulation. These functional assays establish the transcription factor–TPS relationships, whilst the observed methylation changes remain associated with, rather than proven to cause, the corresponding transcriptional changes. In cultivated strawberry (*Fragaria* × *ananassa*), ripening in cv. ‘Hongjia’ was accompanied by reduced RdDM activity, genome-wide DNA hypomethylation and lower 24-nt siRNA abundance. Chemical inhibition of DNA methylation and silencing of ARGONAUTE4 (AGO4), a core component of the RdDM pathway, accelerated ripening, supporting functional involvement of RdDM in the ripening programme; however, the study did not directly test a VOC-biosynthetic locus or volatile output (Cheng et al., 2018). In cv. ‘Yuexin’, a more direct link to aroma metabolism was observed for FaQR3, a quinone oxidoreductase involved in furanone biosynthesis. Reduced FaQR3 promoter methylation was associated with increased gene expression and furanone accumulation. Perturbation of FaAGO4 and the DNA demethylases FaDML1 and FaDML2 altered FaQR3 promoter methylation, FaQR3 expression and furanone accumulation, supporting opposing contributions of RdDM and active demethylation to this pathway (Li et al., 2023).

#### Stress- and post-harvest-associated DNA methylation dynamics

Stress and post-harvest treatments often alter VOC metabolism and DNA methylation simultaneously, complicating causal interpretation. In tea (*Camellia sinensis*), continuous wounding reduced DNA methylation and H3K9me2 at the promoter of CsTSB2, which encodes tryptophan synthase β-subunit 2 and contributes to indole biosynthesis. These changes were accompanied by increased binding of CsMYC2a, a bHLH transcription factor, increased CsTSB2 expression and enhanced indole accumulation. CsDRM3, a DNA methyltransferase involved in *de novo* methylation, was identified as a negative regulator of CsTSB2 expression. Together, these results support a model in which reduced promoter methylation and H3K9me2 increase access of CsMYC2a to the CsTSB2 promoter, thereby promoting indole biosynthesis (Yang et al., 2021). In *Brassica rapa*, feeding by *Pieris brassicae* caterpillars reduced DNA methylation in leaves and flowers and was accompanied by changes in floral volatile composition and reduced attractiveness to pollinators. Direct methyl jasmonate treatment also reduced DNA methylation but produced a partially different volatile profile. Genome-wide demethylation with 5-azacytidine further altered floral scent and reduced pollinator attractiveness in a genotype-dependent manner. These findings support a functional role for DNA methylation in the regulation of floral signalling, although the specific methylation changes responsible for the herbivory-induced volatile response were not identified (Kellenberger et al., 2016). Tomato flavour-associated volatiles are sensitive to post-harvest chilling. In red-ripe *Solanum lycopersicum* cv. ‘Ailsa Craig’, the multi-omics analysis compared fruit after 8 d at 5 °C with fruit exposed to 7 d at 5 °C followed by 1 d recovery at 20 °C (Zhang et al., 2016). Chilling reduced the abundance of multiple flavour volatiles and altered expression of ripening regulators and volatile-biosynthetic genes. These transcriptional changes were accompanied by transient changes in promoter DNA methylation, including at the ripening transcription factors RIN (RIPENING INHIBITOR), NOR (NON-RIPENING) and CNR (COLORLESS NON-RIPENING). Because DNA methylation was not specifically manipulated at these loci, the study supports an association between chilling-induced methylation changes and aroma loss rather than a causal epigenetic mechanism. In peach (*Prunus persica* cv. ‘Hujingmilu’), storage at 0 °C progressively reduced volatile abundance whilst genome-wide DNA methylation changed dynamically during chilling. At individual loci, promoter hypermethylation was detected at PpAAT1, encoding an alcohol acyltransferase involved in ester and lactone biosynthesis; PpTPS3, encoding a linalool synthase; and PpMADS2, a MADS-box transcription factor regulating PpAAT1. These methylation changes were associated with reduced gene expression and lower accumulation of volatile esters, lactones and linalool (Duan et al., 2023). Chilling of *Rosa* ‘Crimson Glory’ flowers reduced expression of several scent-related genes and floral volatile emission. Increased CHH methylation was detected in promoter or genic regions of RhGDS, encoding germacrene D synthase, RhNUDX1, encoding Nudix hydrolase 1 involved in geraniol biosynthesis, and RhNES, encoding nerolidol synthase, and coincided with reduced expression of these genes (Xie et al., 2023). In tea (*C. sinensis*), cold stress was associated with reduced DNA methylation of CsMYB68 and CsMYB147, two MYB21-like transcription factors that act as positive regulators of linalool biosynthesis. Together with the jasmonate-responsive transcription factor CsMYC2, they activate CsTPS76, which encodes a linalool synthase. Reduced methylation was accompanied by increased CsMYB68 and CsMYB147 expression and enhanced linalool accumulation. Treatment with the DNA-methylation inhibitor 5-azacytidine further increased expression of CsMYB68, CsMYB147 and CsTPS76 and increased linalool accumulation, supporting functional involvement of DNA methylation in this pathway (Yue et al., 2025).

During post-harvest oolong tea processing, integrated methylome, transcriptome and metabolome analyses showed that hypomethylation of NES, encoding nerolidol synthase, and AFS1, encoding α-farnesene synthase, coincided with increased expression of these genes and accumulation of nerolidol and α-farnesene. Similar hypomethylation-associated upregulation was observed for several lipid-metabolism genes, whereas methylation–expression relationships in the flavonoid pathway were more heterogeneous. These results further indicate that the transcriptional consequences of DNA methylation depend on gene and pathway context (Kong et al., 2023).

DNA methylation is associated with transcriptional and metabolic differences in VOC-related pathways across developmental, genotypic, stress and post-harvest contexts. Functional perturbation of DNA-methylation machinery supports a regulatory role in several systems, but locus-specific epigenetic causality has not yet been demonstrated.

### Histone modifications

Histone modifications constitute a chromatin-level regulatory layer associated with developmental, diurnal and stress-responsive changes in gene expression. Both histone modifications and DNA methylation can be transient or persistent, depending on the locus and biological context. Histone acetylation is particularly dynamic because acetylation levels are controlled by the opposing activities of histone acetyltransferases and histone deacetylases. Acetylation is generally associated with increased chromatin accessibility, whereas repressive methylation marks such as H3K9me2 and H3K27me3 are commonly associated with transcriptionally repressed chromatin. Other methylation states, including H3K4 and H3K36 methylation, are more frequently associated with transcriptionally active regions, although their regulatory consequences depend on genomic context (Le et al., 2025).

#### Developmental and diurnal regulation of floral VOC biosynthesis

In petunia (*Petunia hybrida* cv. ‘Mitchell’), developmental and day–night changes in floral VOC biosynthesis are associated with dynamic histone acetylation at VOC-related loci. During corolla maturation, increased H3K9ac was detected at genes involved in precursor metabolism and phenylpropanoid/benzenoid VOC biosynthesis, coinciding with increased transcription and volatile emission. Pharmacological inhibition supported functional involvement of histone acetylation, although the effects were inhibitor-dependent: MB-3 reduced H3K9ac and attenuated induction of the tested VOC-related genes, whereas C646 did not alter their transcriptional activation. Transient RNA interference targeting ELP3, which encodes Elongator complex subunit 3 with histone acetyltransferase activity, reduced expression of multiple VOC-related genes and volatile emission (Patrick et al., 2021). A subsequent day–night time-course study showed rhythmic changes in H3K9ac and H3K27ac at VOC-related loci, and inhibition of histone acetylation during the day impaired their normal evening transcriptional induction, further supporting functional involvement of histone acetylation in diurnal regulation of floral VOC metabolism (Patrick et al., 2024).

#### Histone modifications during fruit ripening and post-harvest responses

During fruit ripening, changes in chromatin state accompany the activation of genes involved in volatile ester biosynthesis. Cao et al. (2021) identified conserved transcriptional modules in peach, tomato and apple in which NAC-family transcription factors activate genes encoding alcohol acyltransferases, enzymes catalysing a final step in volatile ester formation. These modules comprise PpNAC1 and PpAAT1 in peach, the NON-RIPENING transcription factor NOR and SlAAT1 in tomato, and MdNAC5 and MdAAT1 in apple. Functional perturbation established the NAC transcription factor–alcohol acyltransferase regulatory relationships. During ripening, activation of these loci also coincided with increased chromatin accessibility and reduced levels of the repressive H3K27me3 mark. Because H3K27me3 itself was not experimentally manipulated, the data support an association between histone state and activation of the ester-biosynthetic modules rather than demonstrating that removal of H3K27me3 is the causal trigger. In cold-stored ‘Nanguo’ pear (*Pyrus ussuriensis*), direct methyl jasmonate treatment increased expression of PuMYC2, a jasmonate-responsive transcription factor. PuMYC2 directly bound the promoter of PuCXE15, which encodes a carboxylesterase involved in volatile ester catabolism, and repressed its transcription. This response was accompanied by reduced histone acetylation at the PuCXE15 promoter and increased accumulation of ester volatiles during storage (Qi et al., 2025). Functional assays support the PuMYC2–PuCXE15 transcriptional module, but histone acetylation at this locus was not directly perturbed.

A separate study in ‘Nanguo’ pear identified a PuWRKY7–PuHDAC15 complex that represses PuAAT1, a gene involved in volatile ester biosynthesis (Zang et al., 2026). PuWRKY7 binds the PuAAT1 promoter, whilst repression by the complex is accompanied by reduced histone acetylation, PuAAT1 expression and ester accumulation. Unlike the PuMYC2–PuCXE15 module, which regulates ester catabolism, this mechanism acts on ester biosynthesis. These findings support functional involvement of a defined transcription factor–HDAC complex, although locus-specific causality of the histone modification itself remains unresolved.

In apple, treatment with a histone deacetylase inhibitor mixture altered the volatile profile, increasing alcohols and aldehydes whilst reducing esters, and also affected cuticular wax metabolism (Song et al., 2026).

#### Stress- and herbivory-associated histone regulation of VOCs

In tea (*Camellia sinensis* ‘Jinxuan’), infestation by the tea green leafhopper (*Empoasca onukii*) affected CsMYC2, the histone deacetylase CsHDA2, and CsNES, which encodes (E)-nerolidol synthase. Infestation increased CsMYC2 binding to the CsNES promoter whilst reducing CsHDA2 expression, protein abundance and promoter occupancy. These changes were accompanied by increased histone acetylation, CsNES expression and (E)-nerolidol accumulation. Histone-deacetylase inhibitor experiments supported a functional role for deacetylation in this response (Gu et al., 2022). During oolong tea processing, mechanical wounding reduced occupancy of the candidate histone deacetylase CsHDA8 at the CsTSB2 promoter. This was accompanied by increased H3 and H4 acetylation, CsTSB2 expression and indole accumulation. Histone-deacetylase inhibitor treatment supported a functional role for deacetylation in this response (Zhou et al., 2022).

Functional evidence is strongest for histone acetylation and deacetylation, whereas H3K27me3 loss during fruit ripening and acetylation changes at PuCXE15 remain correlative.

### Non-coding RNAs as a post-transcriptional regulatory layer

Most non-coding RNA mechanisms currently linked to VOC biosynthesis act at the post-transcriptional level and should not, by themselves, be classified as chromatin-level epigenetic regulation. MicroRNAs (miRNAs) can reduce target-gene expression through transcript cleavage or translational repression, whereas long non-coding RNAs (lncRNAs) can influence transcript processing, stability or miRNA activity (Yu et al., 2017; Wierzbicki et al., 2021). A mechanistically distinct exception is RNA-directed DNA methylation, in which small RNAs participate directly in establishing DNA methylation, as discussed above (Erdmann and Picard, 2020; Wierzbicki et al., 2021). Across the studies reviewed here, transcriptomic, small-RNA and degradome approaches have identified candidate ncRNA–mRNA relationships associated with VOC-related pathways, but only a subset has been functionally validated at the level of target regulation or volatile production. The strength of evidence therefore varies substantially amongst the available ncRNA–VOC studies.

#### Developmental and species-specific ncRNA regulation of floral VOCs

In rose-scented geranium (*Pelargonium graveolens*), transcriptome analysis identified candidate lncRNA–miRNA–mRNA networks associated with terpene biosynthesis, including lncRNAs predicted to act as miRNA precursors or target mimics. Expression of selected lncRNAs was confirmed by quantitative reverse-transcription PCR, but the predicted regulatory interactions were not functionally tested. The study therefore defines candidate ncRNA regulatory networks rather than demonstrated control of terpene biosynthesis (Narnoliya et al., 2019). In *Rosa rugosa*, comparative microRNA profiling and degradome analysis identified post-transcriptional regulatory relationships associated with the mevalonate, methylerythritol phosphate and 2-phenylethanol biosynthetic pathways. Selected microRNA–target interactions were experimentally validated, including miR535f targeting RrHMGR, which encodes 3-hydroxy-3-methylglutaryl-CoA reductase, and the Rosa-specific NOV146 targeting RrNUDX1, which encodes a Nudix hydrolase involved in cytosolic geraniol biosynthesis. Weaker NOV146-mediated repression of RrNUDX1 in the more strongly scented cultivar was associated with greater fragrance production. Other inferred regulatory relationships remain less functionally resolved (Wei et al., 2024). Genome-wide lncRNA profiling in jasmine (*Jasminum sambac*) identified developmentally regulated lncRNAs and predicted targets associated with terpenoid and phenylpropanoid/benzenoid pathways, including terpene synthase genes involved in floral scent formation. These relationships were inferred from enrichment and co-expression analyses and were not functionally validated (Lu et al., 2023).

#### ncRNA-mediated regulation of terpene biosynthesis pathways across taxa

In *Hedychium coronarium*, hco-miR167n directly targets HcARF8, an auxin-response factor that negatively regulates floral-fragrance metabolism. HcARF8 bound the HcTPS8 promoter and reduced its activity in dual-luciferase assays, supporting direct repression of this terpene synthase gene. HcARF8 silencing also affected other fragrance-related genes and volatiles, indicating broader effects beyond the HcARF8–HcTPS8 interaction (Wang et al., 2025). By contrast, transcriptome mining across *Lamiaceae* species identified miRNAs with predicted targets in terpenoid biosynthesis, including 3-hydroxy-3-methylglutaryl-CoA reductase, several terpene synthases, and transcription factors linked to sesquiterpenoid and diterpenoid metabolism. These predictions have not been experimentally validated (Malekroudi et al., 2025). In *Citrus reticulata* cv. ‘Shatangju’, miR2977 post-transcriptionally represses SAMT, which encodes salicylate O-methyltransferase involved in methyl salicylate biosynthesis. Infection with ‘*Candidatus Liberibacter asiaticus*’ reduced miR2977 abundance, increasing SAMT expression and methyl salicylate production and emission. Increasing miR2977 abundance reduced SAMT expression and disease tolerance, whereas SAMT overexpression increased methyl salicylate production. Methyl salicylate also acted as an airborne signal, inducing defence responses in neighbouring citrus plants in controlled exposure and emitter–receiver experiments (Cheng et al., 2023).

### Epitranscriptomic regulation of VOC biosynthesis

N6-methyladenosine (m^6^A) is an epitranscriptomic RNA modification that can affect transcript stability, translation and processing, with its dynamics controlled by RNA methyltransferases, demethylases and m^6^A-binding reader proteins (Hu et al., 2019; Shoaib et al., 2022). Evidence for m^6^A-dependent regulation of VOC biosynthesis has been obtained in tea and tomato. In tea (*Camellia sinensis* cv. ‘Tieguanyin’), CsALKBH4A and CsALKBH4B, which encode m^6^A RNA demethylases, regulated the stability and abundance of transcripts associated with terpenoid biosynthesis during solar withering. Silencing either demethylase increased m^6^A levels and reduced the accumulation of several volatile terpenoids, supporting functional epitranscriptomic involvement in this response (Zhu et al., 2023). In tomato (*Solanum lycopersicum* cv. ‘Ailsa Craig’), two m^6^A reader proteins regulate distinct aroma-related pathways. SlYTH2 binds m^6^A-modified transcripts of SlHPL and SlCCD1B, which encode enzymes contributing to fatty acid- and carotenoid-derived volatile formation, respectively, and represses their translation. Loss of SlYTH2 increased the corresponding aroma volatiles, providing functional evidence for m^6^A-dependent translational regulation (Bian et al., 2024). By contrast, SlYTH1 binds m^6^A-modified SlBCAT1 and SlTNH1 transcripts, which encode key enzymes involved in 2-isobutylthiazole biosynthesis, and promotes their transcript stability and translation. Loss of SlYTH1 consequently reduced 2-isobutylthiazole accumulation during fruit ripening (Gao et al., 2025). Current evidence is limited to VOC-producing tissues, and it is not yet known whether m^6^A also acts downstream of airborne VOC perception.

## From volatile perception to regulatory integration of defence responses

Volatile cues can trigger physiological and transcriptional responses in distal tissues and neighbouring plants, some of which persist after cue removal or alter responses to a subsequent challenge. Receiver-side studies have reported changes in DNA methylation or histone acetylation after exposure to herbivore-induced volatile blends, (E)-β-ocimene and mint volatiles (Ali et al., 2013; Sukegawa et al., 2018; Onosato et al., 2022). Here, persistent phenotypic or chromatin changes after cue removal are distinguished from stress memory, which requires an altered response to a subsequent challenge after a recovery period.

The studies below represent five experimental contexts: (i) regulation of VOC biosynthesis and emission in the producing plant; (ii) emitter–receiver experiments using naturally emitted VOCs or blends; (iii) controlled airborne exposure to defined volatile compounds; (iv) direct application of VOC-related compounds or hormones; and (v) stress or post-harvest treatments in which signalling, regulatory state and VOC production change concurrently. These designs support different mechanistic conclusions. Emitter–receiver and controlled airborne-exposure experiments directly test responses to volatile cues, whereas direct application bypasses airborne perception. Stress and post-harvest studies can link regulatory mechanisms with VOC-related responses but cannot by themselves establish VOC perception as the initiating event. **Table 2** summarises representative studies across these experimental classes. **Supplementary Table S1** provides a study-level matrix covering regulatory components, exposure conditions, early signalling, chromatin- or RNA-level changes, persistence or priming, and evidence strength.

**Table 2:** Experimental contexts and levels of mechanistic evidence in studies linking VOC biology with chromatin-level and RNA-level regulation.

Experimental context (class)	Representative system(s)/cue(s)	Regulatory endpoint(s)	Functional manipulation(s)	Evidence category	What the design supports/key limitation	Representative refs.
(i) Developmental, genotypic and ripening contexts in VOC-producing plants	*Petunia* anthesis and day-night cycle; floral development/cultivar comparisons in *Prunus mume* and *Osmanthus fragrans*; *Jasminum sambac* flower opening; ripening in peach, tomato, apple and strawberry; floral ncRNA and fruit m^6^A studies	Histone acetylation; DNA methylation; chromatin accessibility; RdDM and active demethylation; ncRNA and m^6^A regulation	HAT inhibition and ELP3 knockdown; FaAGO4/FaDML manipulation and 5-azacytidine; STTM/VIGS; CRISPR knockout of m^6^A readers	Association; Functional involvement; RNA-level evidence	Tests regulation of VOC biosynthesis and emission in producing tissues, but does not directly address receiver-side VOC perception or subsequent epigenetic change.	(Cheng et al., 2018; Bao et al., 2019; Narnoliya et al., 2019; Zhang et al., 2019; Cao et al., 2021; Patrick et al., 2021; Wei et al., 2021; Yuan et al., 2021; Han et al., 2022; Wei et al., 2022; Li et al., 2023; Lu et al., 2023; Bian et al., 2024; Patrick et al., 2024; Wei et al., 2024; Gao et al., 2025; Malekroudi et al., 2025; Wang et al., 2025; Zhang et al., 2026)
(ii) Emitter–receiver exposure to naturally emitted VOCs or blends	Maize receivers exposed to HIPVs; mint–soybean co-cultivation; undamaged barley neighbours; SAMT-overexpressing citrus; herbivore-damaged Arabidopsis emitters (GLR-dependent blends); field birch–Arabidopsis exposure.	Promoter DNA methylation; histone acetylation; receiver transcription, Ca^2+^ signalling and pathogen resistance; emitter-side ncRNA regulation.	Garcinol in soybean; SAMT overexpression in citrus; emitter/receiver glr3.3 glr3.6 mutants; high-isoprene birch lines and receiver llp1/jar1 mutants.	Signalling only; Association; Functional involvement; RNA-level evidence	Directly tests airborne plant–plant signalling. Kinoshita (Kinoshita et al., 2026) and field study Zhu (Zhu et al., 2025) show signalling/defence without chromatin endpoints; mixtures prevent attributing all responses to one VOC. Maize methylation was not functionally manipulated.	(Ali et al., 2013; Sukegawa et al., 2018; Cheng et al., 2023; Zhu et al., 2025; Åbonde et al., 2026; Kinoshita et al., 2026)
(iii) Controlled airborne or gaseous exposure to a defined compound	(E)-β-ocimene; α-/β-pinene; (Z)-3-hexenyl butyrate; green leaf volatiles; β-cyclocitral; defined VOC priming before heat challenge; ethylene gas (canonical hormone system).	Ca^2+^, ROS and MAPK signalling; histone acetylation; CHH methylation; pri-miRNAs; heat survival and chromatin-regulator genotype effects.	HAT/HDAC mutants or inhibitors; signalling inhibitors and mpk3/mpk6; ddm1, nrpd1, suvh456 and hda6; haf2; CMT2/EIN3A VIGS and 5-azacytidine.	Signalling only; Association; Functional involvement; RNA-level evidence	Defined airborne exposure tests VOC effects. Barbaruah (Barbaruah et al., 2026) tests chromatin regulators in VOC-primed heat tolerance but does not directly measure VOC-induced chromatin states; the public ChIP-seq comparison does not establish locus-level causality. Ethylene is a canonical hormone model.	(Asai et al., 2009; Riedlmeier et al., 2017; Barczak-Brzyżek et al., 2022; Onosato et al., 2022; Aratani et al., 2023; Payá et al., 2023; Chen et al., 2025; Zhang et al., 2025; Barbaruah et al., 2026)
(iv) Direct application of VOC-related compounds or hormones	JA/MeJA treatments in Norway spruce, Arabidopsis, rice, pear and perennial ryegrass; direct MeSA application in citrus; MeJA in Brassica.	DNA methylation/demethylation machinery; H3K9ac, H4ac and H3K27me3; global methylation; miRNA networks	RdDM, ROS1 and AGO1 genetics; hda6 mutant	Association; Functional involvement; RNA-level evidence	Tests compound-responsive regulatory pathways but bypasses airborne perception. Chromatin states are often measured rather than directly manipulated.	(Kellenberger et al., 2016; Bertini et al., 2018; Wilkinson et al., 2021; Vincent et al., 2022; Wilkinson et al., 2022; Cheng et al., 2023; Liao et al., 2023; Wilkinson et al., 2023; Qi et al., 2025)
(v) Stress and post-harvest treatments with concurrent VOC and regulatory changes	Herbivory; mechanical wounding; chilling of tomato, peach, rose and tea; oolong-tea processing; solar withering; AOX/HDAC manipulation during Nanguo pear ripening; HDAC-inhibitor treatment of stored apples.	DNA methylation; H3K9me2; H3/H4 acetylation and HDAC occupancy (pear); wax/aroma transcript and metabolite changes without locus-resolved chromatin assay (apple); m^6^A.	AOX inhibitor (SHAM), pyruvate, sodium butyrate and PuHDAC15/PuWRKY7 overexpression or antisense silencing in pear; HDAC-inhibitor mixture and MdMYB96 wax-gene assays in apple; 5-azacytidine and m^6^A RNA-demethylase silencing in other systems.	Association; Functional involvement; RNA-level evidence	Post-harvest interventions test regulators of VOC production, not airborne perception. Pear (Zang et al., 2026) links an AOX–TF–HDAC module and PuAAT1 promoter acetylation with ester output but lacks independent locus-specific histone editing. Apple (Song et al., 2026) links HDACi to the volatile profile; MdMYB96 manipulation directly addresses wax rather than aroma VOCs. Chilling studies often remain associative.	(Kellenberger et al., 2016; Zhang et al., 2016; Yang et al., 2021; Gu et al., 2022; Zhou et al., 2022; Duan et al., 2023; Kong et al., 2023; Xie et al., 2023; Zhu et al., 2023; Yue et al., 2025; Song et al., 2026; Zang et al., 2026)

Experimental classes: (i) developmental, genotypic or ripening contexts in VOC-producing plants without external treatment; (ii) emitter–receiver exposure to naturally emitted VOCs or blends; (iii) controlled airborne or gaseous exposure to a defined compound; (iv) direct application of a VOC-related compound or hormone; and (v) stress or post-harvest treatments in which signalling, regulatory state and VOC output change concurrently. Studies with verified designs are assigned a primary class, with additional experimental elements noted where relevant. Gaseous ethylene studies are included in class (iii) but are treated as canonical hormone-signalling systems rather than general models of plant–plant VOC perception.

Evidence categories: Signalling only = VOC-responsive signalling or phenotype without a chromatin or DNA-methylation endpoint; Association = a chromatin or DNA-methylation change coincides with a transcriptional or metabolic outcome without direct manipulation of the regulatory state; Functional involvement = genetic or pharmacological manipulation of chromatin or DNA-methylation machinery alters the response; Locus-specific causality = targeted manipulation of a defined chromatin or DNA-methylation state at a specific locus, not demonstrated in the studies reviewed here; RNA-level evidence = post-transcriptional or epitranscriptomic evidence, not classified as chromatin-level epigenetics.

Interpretive note: No study reviewed here demonstrates locus-specific epigenetic causality through targeted editing of a defined chromatin or DNA-methylation state. Persistent regulatory or phenotypic changes are not classified as stress memory unless a recovery-and-challenge design shows an altered subsequent response.

### Volatile perception, early cellular defence responses, and potential links to chromatin regulation

Volatile cues can induce physiological and transcriptional responses in distal tissues and neighbouring plants, although their perception mechanisms remain unresolved for many compounds (Bergman et al., 2025). In barley (*Hordeum vulgare* L.), volatiles released by undamaged plants altered biomass allocation and growth- and defence-related gene expression in neighbouring plants, with responses depending on the emitter–receiver cultivar combination (Åbonde et al., 2026). The possible contribution of constitutive VOCs to growth–defence adjustments has also been discussed, although the underlying regulatory mechanisms remain unresolved (Engelberth, 2026). Stress-induced volatiles can also activate defence responses (Brosset and Blande, 2022). In *Arabidopsis thaliana*, exposure to α-pinene and β-pinene induced reactive oxygen species accumulation and systemic acquired resistance (Riedlmeier et al., 2017). In tomato, (Z)-3-hexenyl butyrate induced stomatal closure through Ca^2+^ signalling and, in part, NADPH oxidase-derived reactive oxygen species, independently of abscisic acid biosynthesis. The compound also activated MPK3 and MPK6; inhibitor studies and *Arabidopsis mpk3*/*mpk6* mutants implicated these kinases in stomatal immunity. Controlled airborne exposure increased resistance to *Pseudomonas syringae*, whereas the individual signalling components were resolved mainly by direct-application assays (Payá et al., 2023).

Real-time imaging revealed calcium signalling and *VSP1* reporter responses in *Arabidopsis* receivers exposed to herbivore-damaged plants lacking detectable green leaf volatiles, as the Col-0 background lacks functional hydroperoxide lyase (Kinoshita et al., 2026). The glutamate receptor-like (GLR) ion channels GLR3.3 and GLR3.6 were required in emitter plants for the herbivory-induced accumulation of acetophenone, octanal and several alkanes in the headspace and for normal receiver responses, whereas in receiver plants they were required for calcium signalling. The individual contributions of these volatile compounds to the receiver response were not established.

Ca^2+^ transients and reactive oxygen species production are early responses to several plant volatiles (Asai et al., 2009; Riedlmeier et al., 2017; Aratani et al., 2023; Payá et al., 2023), but their role in subsequent chromatin regulation has not been established. Outside VOC signalling, H_2_O_2_-associated S-sulfenylation of the *Arabidopsis* histone acetyltransferase GCN5 shows that redox status can directly modify a chromatin-regulatory protein, but does not establish a VOC-induced redox-to-chromatin pathway (De Smet et al., 2025). Chromatin changes after airborne volatile exposure have nevertheless been reported in receiver plants, including promoter DNA-methylation changes in maize and histone-acetylation changes in soybean and *Arabidopsis* (Ali et al., 2013; Sukegawa et al., 2018; Onosato et al., 2022). **Figure 3** summarises these signalling and regulatory steps and highlights the mechanistic links that remain to be resolved.

**Figure 3:**
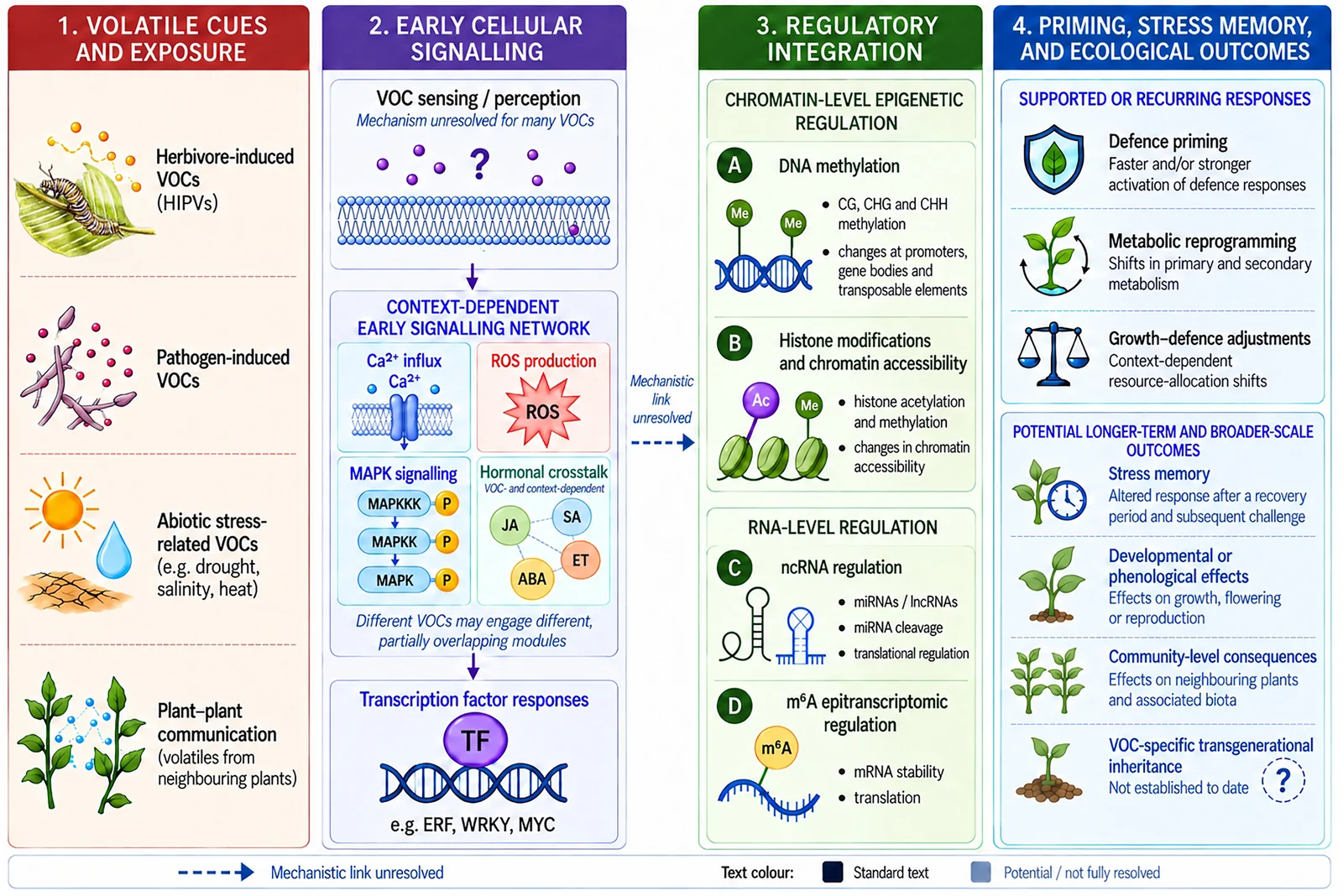
From volatile cue perception to regulatory integration and longer-term plant responses. Plant VOCs can activate partially overlapping signalling pathways involving Ca^2+^, ROS, MAPKs, hormonal crosstalk and transcription-factor responses. These signals may connect with chromatin- and RNA-level regulation, although the underlying mechanisms remain incompletely resolved. VOC exposure can be associated with defence priming, metabolic reprogramming and context-dependent growth–defence adjustments. VOC-specific transgenerational inheritance has not been established. Dashed arrows indicate unresolved mechanistic links, and muted text denotes potential or incompletely resolved outcomes.

### DNA methylation in volatile-induced and related stress responses

The clearest direct evidence linking airborne VOC exposure to DNA-methylation changes in receiver plants comes from maize emitter–receiver experiments (Ali et al., 2013). Related studies using jasmonate application or gaseous ethylene address DNA-methylation machinery in defence and hormone signalling, but do not directly test plant–plant VOC perception (Bertini et al., 2018; Wilkinson et al., 2022; Wilkinson et al., 2023; Zhang et al., 2025).

Exposure of maize (*Zea mays*) receivers to herbivore-induced volatiles from infested conspecifics primed subsequent anti-herbivore responses. Enhanced resistance and stronger induction of a Bowman–Birk-type trypsin inhibitor gene persisted after a post-exposure recovery period. Bisulfite analysis also revealed demethylation at selected cytosines in the trypsin inhibitor promoter. However, methylation was not followed throughout recovery or experimentally manipulated, so its role in maintaining the primed response remains unclear (Ali et al., 2013). In Norway spruce (*Picea abies*), methyl jasmonate treatment induced prolonged changes in expression of components of RNA-directed DNA methylation and active demethylation during long-term induced resistance. DNA methylation itself was not measured, so the study documents sustained regulation of methylation machinery rather than persistent methylation changes (Wilkinson et al., 2022). In *Arabidopsis thaliana*, long-lasting resistance induced by jasmonic acid required components of RNA-directed DNA methylation, the DNA demethylase REPRESSOR OF SILENCING 1 (ROS1) and ARGONAUTE1 (AGO1) and was associated with selective hypomethylation of ATREP2 transposable elements. Because jasmonic acid was applied directly, this experiment does not test airborne VOC perception (Wilkinson et al., 2023). In rice (*Oryza sativa*), direct methyl jasmonate pretreatment followed by mechanical wounding primed expression of OsBBPI, which encodes a Bowman–Birk protease inhibitor, and OsPOX, which encodes a wound-inducible peroxidase. This response was accompanied by altered histone acetylation at the OsBBPI promoter and changes in global DNA methylation, whereas the examined CpG islands in the OsBBPI and OsPOX promoters remained unchanged (Bertini et al., 2018). This supports chromatin involvement in priming but not locus-specific DNA-methylation control of these genes. In non-heading Chinese cabbage (*Brassica rapa*), the ethylene-responsive transcription factor EIN3A repressed CMT2, which encodes a CHROMOMETHYLASE DNA methyltransferase. Reduced CMT2-dependent CHH methylation was associated with increased EIN3A occupancy and activation of senescence-associated genes. Genetic analyses and 5-azacytidine treatment further implicated DNA-methylation machinery in this response (Zhang et al., 2025). This links gaseous hormone signalling with chromatin regulation, but does not address plant–plant VOC perception.

The maize emitter–receiver study directly links airborne volatile exposure with promoter demethylation, whereas jasmonate and ethylene models implicate DNA-methylation machinery in persistent or primed responses. None demonstrates locus-specific epigenetic causality for VOC-induced priming.

### Histone modifications in volatile-induced signalling

Direct evidence that airborne volatile exposure can alter histone acetylation in receiver plants comes from controlled β-ocimene exposure in *A. thaliana* and mint–soybean emitter–receiver experiments (Sukegawa et al., 2018; Onosato et al., 2022). β-Ocimene exposure increased histone acetylation at ERF8 and ERF104, which encode AP2/ERF-family transcription factors involved in defence responses, and enhanced resistance to *Spodoptera litura*. The anti-herbivore response persisted for up to 5 d after exposure but was no longer detectable after 10 d. Treatment with the histone acetyltransferase inhibitor garcinol suppressed β-ocimene-induced ERF8 and ERF104 expression. Mutant analyses further implicated HAC1, HAC5 and HAM1 in histone acetylation at these loci, whilst *hac1*, *hac2*, *hac5* and *ham1* mutants failed to show the normal β-ocimene-induced anti-herbivore response. HDA6 contributed to reversal of the acetylated state (Onosato et al., 2022). These experiments establish a functional role for histone acetylation in the β-ocimene response, although its requirement for persistence of that response was not directly tested. Direct methyl jasmonate treatment in *Arabidopsis* also altered chromatin regulation. ChIP-seq analysis of wild-type and *hda6* (axe1-5) plants treated with or without methyl jasmonate identified both MeJA- and HDA6-dependent changes in H4 acetylation and H3K27 trimethylation at genes involved in stress responses and specialised metabolism (Vincent et al., 2022). Because methyl jasmonate was applied directly, this study informs jasmonate–chromatin regulation but does not test airborne VOC perception. In a separate ethylene model, HAF2 interacted with the C-terminal region of ETHYLENE INSENSITIVE 2 (EIN2-C) in response to ethylene. Ethylene-induced H3K14 and H3K23 acetylation and activation of target genes were impaired in *haf2* mutants (Chen et al., 2025). This establishes a link between canonical ethylene signalling and chromatin regulation but should not be generalised to other plant VOCs. In soybean (*Glycine max*), co-cultivation with candy mint increased expression of the defence genes TI, encoding a trypsin inhibitor, and PR1, encoding PATHOGENESIS-RELATED 1, together with histone acetylation at their promoters. After a 7-d exposure, transcript levels remained elevated for up to 8 d after removal of the mint source. Promoter acetylation was also elevated immediately and 8 d after source removal, but returned to control levels by 12 d. The histone acetyltransferase inhibitor garcinol attenuated the volatile-induced transcriptional response (Sukegawa et al., 2018). At greater emitter–receiver distances, basal TI and PR1 expression was unchanged, whereas subsequent herbivore challenge revealed primed responses. Because the chromatin time course was measured only in the proximate co-cultivation system, histone acetylation was not directly linked to the distance-dependent priming response.

Chromatin regulators acting at both DNA-methylation and histone levels have also been examined in VOC-induced priming. In *Arabidopsis*, a 30-min exposure to defined VOCs followed by 48 h of recovery improved survival after a 30-min heat challenge at 45 °C in wild-type plants and mutants affecting DNA methylation, RNA-directed DNA methylation, H3K9 methylation and histone deacetylation (*ddm1*, *nrpd1*, *suvh456* and *hda6*) (Barbaruah et al., 2026). Responses varied amongst genotypes, with trans-2-hexenal producing a stronger shift towards healthy seedlings in *hda6*. Transcriptomic analysis identified partially overlapping VOC- and heat-responsive programmes, whereas chromatin associations were inferred from previously published ChIP-seq datasets. These findings indicate that chromatin regulators modulate VOC-responsive stress networks, but do not establish that a specific VOC-induced chromatin state is required for priming.

### Non-coding RNA responses to volatile exposure and related hormone treatments

Receiver-side evidence for non-coding RNA responses to airborne VOCs remains limited. In *Arabidopsis*, controlled airborne exposure to β-cyclocitral altered the abundance of selected primary microRNA transcripts, including pri-miR163 and pri-miR840. In the same study, high-light induction of pri-miR163 and pri-miR840 was attenuated in the *mbs1* mutant, consistent with involvement of the β-cyclocitral-dependent singlet-oxygen retrograde pathway (Barczak-Brzyżek et al., 2022). High-light stress, which can promote oxidative β-carotene cleavage and β-cyclocitral formation, also alters a broader range of small RNAs, including microRNAs, trans-acting small interfering RNAs and natural antisense small interfering RNAs (Ramel et al., 2012; Tiwari et al., 2021). However, these broader high-light-induced small-RNA changes cannot be attributed specifically to β-cyclocitral. The airborne β-cyclocitral experiment therefore shows changes in selected pri-miRNAs but does not establish downstream miRNA-mediated regulation of target genes. Related RNA-level responses have been reported after direct methyl jasmonate treatment, which bypasses airborne volatile perception. In Norway spruce, methyl jasmonate application induced prolonged changes in the bark microRNA transcriptome, whilst a subset of microRNAs showed altered responses to subsequent mechanical wounding. Predicted targets included NBS-LRR immune receptors and regulators of jasmonate biosynthesis, but miRNA and predicted target expression showed little overall correspondence (Wilkinson et al., 2021). In perennial ryegrass (*Lolium perenne*), direct methyl jasmonate pretreatment altered miRNA–mRNA networks associated with the subsequent heat-stress response, including pathways related to signal transduction, protein homeostasis and cell-wall metabolism (Liao et al., 2023). These MeJA studies show prolonged or challenge-dependent miRNA responses after direct hormone application, but they do not test ncRNA regulation initiated by airborne VOC exposure.

## Conclusion

In VOC-producing tissues, DNA methylation and histone modifications are associated with VOC biosynthesis and emission, with functional involvement demonstrated in selected systems (Gu et al., 2022; Li et al., 2023; Patrick et al., 2024; Yue et al., 2025). Post-transcriptional ncRNA regulation and m^6^A-dependent mechanisms provide additional RNA-level control of selected VOC biosynthetic pathways (Zhu et al., 2023; Bian et al., 2024; Gao et al., 2025; Wang et al., 2025). By contrast, evidence that airborne VOC perception induces chromatin- or RNA-level regulation in receiver plants is more limited. Controlled airborne exposure and emitter–receiver studies have linked selected VOCs or volatile blends with changes in DNA methylation or histone acetylation, and chromatin regulators have been functionally tested in some systems (Ali et al., 2013; Sukegawa et al., 2018; Onosato et al., 2022). Studies based on direct application of VOC-related compounds or hormones provide useful information on downstream regulation but do not test airborne VOC perception. A general pathway connecting volatile perception with chromatin reprogramming has therefore not been established.

Early responses to several VOCs include Ca^2+^ signalling, ROS production and MAPK-associated signalling (Asai et al., 2009; Riedlmeier et al., 2017; Aratani et al., 2023; Payá et al., 2023), but their connection with subsequent chromatin- or RNA-level regulation remains unclear. Likewise, m^6^A-dependent regulation of VOC biosynthesis is supported experimentally (Zhu et al., 2023; Bian et al., 2024; Gao et al., 2025), whereas its involvement downstream of airborne VOC perception has not been demonstrated. How complex volatile blends are integrated, and whether different tissues or cell types engage distinct regulatory responses to the same volatile environment, also remain open questions.

Persistent and primed responses following volatile exposure have been reported in several systems (Ali et al., 2013; Sukegawa et al., 2018; Onosato et al., 2022), but persistence alone does not establish stress memory. Chromatin regulators have been implicated in VOC-primed thermotolerance (Barbaruah et al., 2026), yet whether a defined VOC-induced chromatin state is required for an altered response after recovery and subsequent challenge remains unresolved (Ali and Moon, 2026; Harris et al., 2023). A general epigenetic mechanism of VOC-induced stress memory and VOC-specific transgenerational inheritance therefore remain unestablished.

Resolving causality will require controlled airborne exposure to defined compounds or chemically characterised blends, time-resolved exposure–recovery–challenge experiments, and direct testing of candidate regulatory mechanisms. Cell-type- and spatially resolved transcriptomic and chromatin approaches can determine where regulatory changes arise (Ouyang et al., 2022; Lu et al., 2024; Sun et al., 2024; Nobori, 2025), whilst locus-specific approaches such as CRISPR/dCas-based epigenome editing can test whether particular chromatin states are required for persistent or primed responses (Papikian et al., 2019; Harris et al., 2023). Recent analyses of tea hybrids have also associated three-dimensional chromatin organisation with expression changes in aroma-related genes (Lei et al., 2026), suggesting a further level for investigation.

Ecological studies show that VOC-mediated interactions can affect neighbouring plants, herbivores and higher trophic levels (Grof-Tisza et al., 2024; Thompson et al., 2025; Medina-van Berkum et al., 2026). In a field experiment, volatile blends from birch lines with elevated isoprene emission increased bacterial resistance in neighbouring *Arabidopsis* plants, although chromatin- and RNA-level regulation was not examined (Zhu et al., 2025). Establishing the ecological significance of these regulatory layers will require linking controlled mechanistic experiments with field-relevant emitter–receiver systems.
