# Supplementary material for: Epigenetic and RNA-mediated regulation of plant VOC biosynthesis and signalling
Source: Front Plant Sci. 2026 Oct 20;17:1945830. doi: 10.3389/fpls.2026.1945830 (PMC13728493; doi:10.3389/fpls.2026.1945830)
Supplement: Supplementary file 1 [file Table1.docx]

**Supplementary Table S1. Evidence matrix for VOC-related chromatin regulation, RNA-level regulation, early signalling, persistence and priming.**

This table summarises the primary studies cited in the review. For each study, it reports the regulatory component, plant system, exposure context, early signalling, chromatin- or RNA-level changes, persistence or priming, temporal design, experimental manipulation, and evidence category. “Not examined” indicates that the cited study did not test that element. RNA-level evidence is considered separately from chromatin-level epigenetic evidence, and persistence alone is not classified as stress memory.

Experimental classes: (i) Developmental, genotypic and ripening contexts in VOC-producing plants; (ii) Emitter–receiver exposure to naturally emitted VOCs or blends; (iii) Controlled airborne or gaseous exposure to a defined compound; (iv) Direct application of VOC-related compounds or hormones; and (v) Stress and post-harvest treatments with concurrent VOC and regulatory changes. Studies labelled “Supporting context” are mechanistic background without an additional class. Post-harvest AOX and HDAC interventions in pear and apple [55, 56] belong to class (v).

| **Gene(s) / regulatory component(s)** | **Function or role** | **Plant species** | **Cue / exposure mode (primary class)** | **Early signalling** | **Chromatin- or RNA-level change(s)** | **Priming / persistence / stress memory** | **Temporal design** | **Assay / functional manipulation** | **Evidence category / key limitation** | **Ref.** |
| --- | --- | --- | --- | --- | --- | --- | --- | --- | --- | --- |
| ELP3; GNAT-family HATs; VOC-network genes | ELP3 is a GNAT-family histone acetyltransferase; the VOC network links phenylalanine supply with phenylpropanoid/benzenoid biosynthesis | *Petunia hybrida* cv. Mitchell | Developmental anthesis; no external VOC exposure [class (i)] | Not examined | H3K9ac increases across precursor-supplying and VOC-biosynthetic genes after anthesis | Developmental regulation; no priming or stress memory test | Pre-anthesis vs open flowers; inhibitor treatment initiated before anthesis. | ChIP-seq/ChIP, expression and VOC analysis. HAT inhibitors C646 and MB-3 and transient ELP3 downregulation were tested; locus-specific chromatin causality was not assessed. | Functional involvement (chromatin) | 20 |
| Floral VOC metabolic network genes | Diurnal control of floral volatile metabolism and precursor supply | *Petunia hybrida* cv. Mitchell | Endogenous day–night cycle; no external VOC exposure [class (i)] | Not examined | Rhythmic H3K9ac and H3K27ac at VOC-related loci, largely removed at night | Diurnal regulation; no priming or stress memory test | Day/night time course with multiple ChIP-qPCR time points. | Time-course ChIP with expression and VOC profiling. MB-3 HAT inhibition tested the role of daytime acetylation. | Functional involvement (chromatin) | 21 |
| PmCFAT1a/1c; PmBEAT36/37; PmPAL2; PmPAAS3; PmBAR8/9/10; PmCNL1/3/5/6/14/17/20 | CFAT, coniferyl alcohol acetyltransferases (eugenol pathway); BEAT, benzyl alcohol O-acetyltransferases (benzyl acetate); PAL, phenylalanine ammonia-lyase; PAAS, phenylacetaldehyde synthase; BAR, benzaldehyde reductases; CNL, cinnamate:CoA ligases | *Prunus mume* | Flower development; no external VOC exposure [class (i)] | Not examined | 22 scent-pathway genes differentially methylated. PmBEAT36 shows promoter CG hypomethylation with increased expression, whereas PmBEAT37 expression increases despite promoter CHH hypermethylation, with gene-body CG hypomethylation | Not examined | Four bud/flower developmental stages. | WGBS + RNA-seq. Shows locus- and context-dependent methylation–expression relationships; DNA methylation was not manipulated. | Association | 37 |
| PmBEAT36; PmBEAT37 | Benzyl alcohol O-acetyltransferases catalysing benzyl acetate formation in petals | *Prunus mume* | Functional gene assay; no VOC exposure and no epigenetic treatment [class (i)] | Not applicable | No epigenetic endpoint measured | Not applicable | Transient petal-protoplast assay. | Petal-protoplast assays with GC-MS. PmBEAT36/37 overexpression and interference validate the enzymatic step but do not test methylation. | Functional gene validation; no epigenetic endpoint | 36 |
| TPS2 (and TPS1) | Terpene synthase of the linalool biosynthetic pathway | *Osmanthus fragrans* | Cultivar comparison (‘Chenghong Dangui’ vs ‘Zaohuang’); no external VOC exposure [class (i)] | Not examined | Lower TPS2 promoter methylation and greater chromatin accessibility coincide with markedly higher TPS2 expression and more linalool and linalool oxides | Not examined | Cultivar comparison at full flowering. | WGBS + ATAC-seq with expression and scent analysis; methylation and chromatin accessibility were not manipulated. | Association | 39 |
| Selected terpene synthase (TPS) genes | Terpenoid floral-scent biosynthesis | *Jasminum sambac* ‘Bijian’ | Flower opening/development; no external VOC exposure [class (i)] | Not examined | Overall 5mC increase from bud to full flowering; higher CHH methylation within selected TPS gene bodies coincides with increased transcript abundance | Not examined | Five flowering stages (FS1–FS5) sampled sequentially at 18:00, 19:30, 21:00, 22:30 and 24:00 on one night; WGBS compared FS1 (bud) with FS4 (full-flowering I). | Volatile metabolomics and transcriptomics across FS1–FS5; WGBS at FS1 and FS4 with BSP validation. Association; developmental stage and sampling time are confounded in this design. | Association | 40 |
| PpERF61; PpTPS1; PpTPS3 | PpERF61 transcriptionally activates the linalool synthases PpTPS1 and PpTPS3 | *Prunus persica* | Fruit ripening; no external VOC exposure [class (i)] | Ripening signalling; not resolved as VOC perception | DNA demethylation at the PpERF61 locus during ripening is associated with increased PpERF61 expression and linalool accumulation; PpERF61 directly activates PpTPS1 and PpTPS3. | Not examined | Fruit ripening series. | Dual-luciferase/EMSA, expression and linalool quantification with PpERF61 overexpression and TF–promoter assays. Establishes TF–TPS regulation; DNA methylation was not manipulated. | Association (methylation); functional TF–target module | 41 |
| PpbHLH1; PpTPS3 | PpbHLH1 regulates the linalool synthase PpTPS3 | *Prunus persica* | Fruit ripening; no external VOC exposure [class (i)] | Ripening signalling; not resolved | Methylation changes accompany the increase in PpTPS3 expression and linalool | Not examined | Fruit developmental series. | WGBS, expression, GC-MS and TF assays. Establishes PpbHLH1–PpTPS3 regulation; DNA methylation was not manipulated. | Association (methylation); functional TF–target module | 42 |
| RdDM machinery (Pol IV/Pol V subunits, DRM/CMT orthologues, AGO4-type component) | Core RdDM pathway controlling DNA methylation during non-climacteric fruit ripening | *Fragaria × ananassa* Duch. cv. ‘Hongjia’ (5-azacytidine assay also used cv. ‘Si gongzhu’; *F. vesca* was the reference genome) | Fruit ripening; no external VOC exposure [class (i)] | Not examined | Ripening-associated downregulation of RdDM, genome-wide DNA hypomethylation and reduced 24-nt siRNA abundance | Not examined | Fruit-ripening series; 5-azacytidine/VIGS experiments during ripening. | WGBS, sRNA-seq and RNA-seq with 5-azacytidine and TRV-mediated AGO4-type silencing. Tests RdDM in ripening, but no VOC locus or volatile output was directly assayed. | Functional involvement in ripening; not VOC-specific | 43 |
| FaQR3; FaAGO4; FaDML1/2 | FaQR3 is the predominant quinone oxidoreductase contributing to furanone biosynthesis; FaAGO4 promotes RdDM; FaDML1/2 mediate active demethylation | *Fragaria × ananassa* cv. ‘Yuexin’ | Fruit ripening and temperature treatments; no external VOC exposure [class (i)] | Not examined | RdDM and active demethylation act antagonistically at the FaQR3 promoter; reduced promoter methylation is accompanied by higher FaQR3 expression and furanone accumulation | Not examined | Fruit-ripening series; FaDML RNAi and methylation-inhibitor time courses. | Promoter bisulfite/24-nt siRNA analyses with FaAGO4 overexpression/RNAi, FaDML1/2 RNAi, 5-azacytidine and furanone quantification. | Functional involvement (RdDM/demethylation) | 44 |
| CsTSB2; CsMYC2a; CsDRM3 | CsTSB2 encodes tryptophan synthase β-subunit 2 and contributes to indole formation; CsMYC2a is a bHLH TF; CsDRM3 is a domains-rearranged DNA methyltransferase | *Camellia sinensis* | Continuous mechanical wounding during oolong-tea processing [class (v)] | Wound-responsive transcriptional signalling; upstream pathway not fully resolved | Reduced DNA methylation and H3K9me2 at the CsTSB2 promoter coincide with increased CsMYC2a binding, CsTSB2 expression and indole production; CsDRM3 is implicated as a negative regulator. | Not examined | Continuous wounding; 6 h endpoint. | Promoter methylation, histone-mark and TF-binding analyses. Reduced methylation/H3K9me2 coincided with increased CsMYC2a binding; the methylation machinery was not directly manipulated. | Association at a defined locus | 45 |
| RIN; NOR; CNR; multiple volatile-biosynthetic genes | Ripening transcription factors and enzymes contributing to tomato aroma | *Solanum lycopersicum* cv. Ailsa Craig | Postharvest chilling at 5 °C [class (v)] | Cold-stress signalling; not resolved in this analysis | Transient promoter DNA-methylation changes accompany reduced expression of ripening regulators and VOC-biosynthetic genes and aroma loss. | Methylation changes partly reversible after chilling and recovery; not tested as priming or stress memory | 8 d at 5 °C, or 7 d at 5 °C followed by 1 d recovery at 20 °C. | WGBS, RNA-seq and volatile/metabolite profiling; DNA methylation was not manipulated at the affected loci. | Association | 47 |
| PpAAT1; PpTPS3; PpMADS2 (also PpACS1, PpERF61) | PpAAT1 contributes to ester and lactone biosynthesis; PpTPS3 is a linalool synthase; PpMADS2 is a transcription factor regulating PpAAT1 | *Prunus persica* L. Batsch cv. Hujingmilu | Postharvest cold storage at 0 °C [class (v)] | Cold-stress signalling; not resolved | Genome-wide DNA methylation increases during chilling, peaks around 7 d and then declines; promoter hypermethylation of PpAAT1, PpTPS3 and PpMADS2 is associated with reduced expression | Total volatiles fall by ~50% at 3 d and ~90% at 28 d; no recovery/challenge design | 0 °C storage time course up to 28 d. | Methylome, transcriptome and GC-MS profiling; the DNA-methylation machinery was not manipulated. | Association | 48 |
| RhGDS; RhNUDX1; RhNES; RhPAAS; RhPAR; RhAAT1 | RhGDS (germacrene D synthase), RhNUDX1 (geraniol-related pathway), RhNES (nerolidol synthase), RhPAAS/RhPAR (aromatic alcohol pathway) and RhAAT1 (alcohol acyltransferase). | *Rosa* ‘Crimson Glory’ | Chilling of cut flowers [class (v)] | Cold-stress signalling; not resolved | Expression of several scent genes decreased after chilling. Locus-level methylation analysis showed increased CHH methylation in promoter/genic regions of RhGDS, RhNUDX1 and RhNES, coinciding with reduced expression and scent emission. | Not examined | 6 °C for 24 h followed by 24 h recovery at 20 °C. | WGBS with expression and volatile profiling; methylation was not manipulated. | Association | 49 |
| CsMYB68; CsMYB147; CsMYC2; CsTPS76 | Divergent MYB paralogues, together with CsMYC2, activate the linalool synthase CsTPS76 | *Camellia sinensis* | Cold stress; JA treatment/processing context [class (v)] | JA signalling increases MYC2–MYB activity | Cold-associated DNA demethylation increases CsMYB68/CsMYB147 expression; 5-azacytidine also increases pathway expression and linalool | Cold-tolerance phenotype assessed; no formal recovery/challenge memory design | Cold-stress time course; 5-azacytidine pretreatment used in functional assays. | TF interaction/binding assays, gene manipulation, metabolite analysis and 5-azacytidine treatment. DNA methylation was implicated in the CsMYB–CsTPS76–linalool pathway under cold stress. | Functional involvement (DNA methylation) | 50 |
| NES; AFS1; lipid-metabolism genes | Nerolidol synthase and α-farnesene synthase; volatile terpenoid production | *Camellia sinensis* | Postharvest withering and turnover during oolong-tea processing [class (v)] | Not resolved | Hypomethylation of NES and AFS1 coincides with increased expression and nerolidol/α-farnesene accumulation; methylation–expression relationships in the flavonoid pathway are heterogeneous | Not examined | Postharvest withering/turnover stages during oolong-tea processing. | Integrated methylome, transcriptome and metabolome analysis; DNA methylation was not manipulated. | Association | 51 |
| PpNAC1–PpAAT1; NOR–SlAAT1; MdNAC5–MdAAT1 | Conserved NAC transcriptional modules activate alcohol acyltransferases catalysing the final step of volatile ester formation | *Prunus persica*; *Solanum lycopersicum*; *Malus domestica* | Fruit ripening; no external VOC exposure [class (i)] | Ripening transcriptional signalling | Increased chromatin accessibility and loss of the repressive H3K27me3 mark at NAC/AAT loci accompany their activation | Not examined | Species-specific fruit-ripening series. | fruitENCODE chromatin and methylation datasets with RNA-seq/VOC profiling. NAC/AAT genes were functionally tested, but H3K27me3 itself was not manipulated. | Association (chromatin); functional TF–target module | 53 |
| PuMYC2; PuCXE15 | JA-responsive TF PuMYC2 represses the carboxylesterase PuCXE15, limiting volatile ester catabolism | *Pyrus ussuriensis* ‘Nanguo’ | Direct MeJA treatment during cold storage [class (iv)] | JA → PuMYC2 transcriptional response | Reduced histone acetylation at the PuCXE15 promoter accompanies its repression | Ester aroma preserved during storage; no recovery/challenge memory design | MeJA treatment followed by long-term cold storage. | TF–promoter binding, promoter-acetylation and ester profiling with MeJA/PuMYC2 manipulation. Establishes transcriptional regulation; promoter acetylation itself was not manipulated. | Association (histone acetylation); functional TF–target module | 54 |
| AOX pathway; PuHDAC15; PuWRKY7; PuAAT1 | AOX promotes ester formation; PuHDAC15 and promoter-binding PuWRKY7 cooperate to repress the alcohol acetyltransferase PuAAT1. | Pyrus ussuriensis ‘Nanguo’ | Post-harvest ripening with direct AOX-pathway and HDAC interventions; no airborne VOC exposure [class (v)]. | AOX-related respiratory/metabolic regulation; VOC perception was not tested. | AOX inhibition reduced H3ac/H4ac at PuAAT1 promoter regions, PuAAT1 expression and ester accumulation; SHAM + sodium butyrate increased acetylation and ester abundance relative to SHAM alone. PuHDAC15/PuWRKY7 co-expression strengthened promoter deacetylation and repression. | Aroma accumulation during ripening; no VOC-initiated recovery–challenge or stress-memory assay. | Fruit harvested at 135 d after full bloom; 20-min treatment with 0.5 mM SHAM, 1 mM pyruvate or water, then 25 °C storage; respiration/aroma sampled every 5 d; PuAAT1 H3ac/H4ac assayed at 10 d after harvest. SHAM + 200 mM sodium butyrate tested separately. | AOX respiration; HS-SPME/GC–MS; RT-qPCR; promoter H3ac/H4ac ChIP-qPCR; PuHDAC15 and PuWRKY7 transient overexpression/antisense silencing; protein interaction (Y2H, pull-down, LCI), promoter binding (Y1H, EMSA) and GUS reporter assays. | Functional involvement of AOX–HDAC–TF machinery in ester biosynthesis with promoter-associated acetylation changes; acetylation at PuAAT1 was not independently edited, and VOC reception/memory was not tested. | 55 |
| HDAC-inhibitor treatment; MdMYB96; wax- and aroma-related genes | Pharmacological HDAC inhibition alters apple aroma and wax metabolism; MdMYB96 activates wax-biosynthetic genes, not proven to control individual VOC changes. | Malus domestica: ‘Red Delicious’ (volatile profiling); ‘Pink Lady’ and ‘Fuji’ (RNA-seq and wax analyses). | Direct injection of an HDAC-inhibitor mixture into harvested fruits during storage; no airborne VOC exposure [class (v)]. | Receiver-side VOC perception and early signalling not examined. | In ‘Red Delicious’, alcohols/aldehydes increased and esters decreased after treatment. Wax-pathway transcripts and MdMYB96 increased in other cultivar assays. No locus-specific histone-mark measurement or selective histone-state manipulation established the VOC mechanism. | Post-harvest aroma/wax responses; no VOC-priming, recovery–challenge or stress-memory test. | At harvest, 1 mL inhibitor mixture or water injected per fruit; storage in darkness at 20 °C and 95% RH; peel/flesh sampled at 0, 1, 3, 6 and 20 d; ‘Pink Lady’/‘Fuji’ RNA-seq at 6 d; ‘Fuji’ wax analysis at 14 d. | HDACi mixture: 40 μM trichostatin A, 1 mM EX-527, 400 mM nicotinamide and 200 mM sodium butyrate (as reported); HS-SPME/GC–MS aroma in ‘Red Delicious’; RNA-seq, wax assays, MdMYB96 promoter/reporter assays, fruit VIGS and Arabidopsis overexpression for wax function. | Pharmacological association/involvement in volatile-profile changes; MdMYB96 gene-function assays concern wax. Mixture may affect non-histone protein acetylation; no defined causal chromatin mark for individual aroma VOCs. | 56 |
| CsMYC2; CsHDA2; CsNES | JA-responsive CsMYC2 and the histone deacetylase CsHDA2 regulate the (E)-nerolidol synthase CsNES | *Camellia sinensis* ‘Jinxuan’ | Tea green leafhopper (*Empoasca onukii*) infestation [class (v)] | JA-associated signalling; increased CsMYC2 binding | Reduced CsHDA2 abundance/binding and increased H3/H4 acetylation at CsNES accompany higher CsNES expression and (E)-nerolidol production | Not examined | Leafhopper infestation with separate JA and HDAC-inhibitor assays. | ChIP, expression, (E)-nerolidol quantification and protein-regulatory assays, including HDAC-inhibitor and JA treatments. Herbivory does not isolate VOC perception. | Functional involvement (histone deacetylation) | 57 |
| CsHDA8; CsTSB2 | CsHDA8 is a candidate histone deacetylase regulator; CsTSB2 contributes to indole biosynthesis | *Camellia sinensis* ‘Jinxuan’ | Mechanical wounding during oolong-tea processing [class (v)] | Wound signalling; upstream pathway not resolved | Reduced CsHDA8 occupancy at the CsTSB2 promoter with elevated H3ac/H4ac and increased CsTSB2 expression | Not examined | Wounding time course (0–6 h) with HDAC-inhibitor pretreatment. | ChIP, expression and indole measurements. HDAC-inhibitor pretreatment increased global H3ac/H4ac but reduced wound-induced CsTSB2 expression and indole accumulation, indicating a non-linear relation between bulk acetylation and the response. Inhibitor cocktail: 0.25 mM each TSA, SBHA, sodium butyrate and nicotinamide. | Functional involvement (histone deacetylation) | 58 |
| Candidate lncRNA–miRNA–mRNA modules | Predicted regulation of terpenoid and related metabolic genes | *Pelargonium graveolens* | Developmental/tissue context; no defined VOC exposure [class (i)] | Not examined | Post-transcriptional ncRNA network; not evidence of chromatin-level regulation | Not examined | Developmental/tissue transcriptome comparison. | Transcriptome network analysis with qRT-PCR validation of selected lncRNAs; predicted regulatory interactions were not functionally tested. | Candidate RNA-level association | 59 |
| miR535f–RrHMGR; NOV146–RrNUDX1; miR166l–RrHY5; miR156c–RrSPL2 | HMGR contributes to the MVA pathway; RrNUDX1 hydrolyses geranyl diphosphate in cytosolic geraniol biosynthesis | *Rosa rugosa* | Cultivar/developmental fragrance comparison; no defined VOC exposure [class (i)] | Not examined | Post-transcriptional miRNA-mediated target regulation; selected cleavage interactions validated. | Not examined | Full-bloom cultivar comparison. | sRNA sequencing, degradome and RLM-RACE validated selected cleavage events; individual miRNA modules were not genetically manipulated. | Validated RNA–target interactions; fragrance contribution partly inferential | 60 |
| Candidate lncRNAs; JsTPS targets | Putative regulation of terpenoid and phenylpropanoid/benzenoid scent pathways | *Jasminum sambac* | Floral development; no defined VOC exposure [class (i)] | Not examined | Predicted lncRNA–target and co-expression relationships | Not examined | Flower buds vs full-bloom flowers. | Genome-wide lncRNA profiling and co-expression analysis; no direct lncRNA manipulation. | Candidate RNA-level association | 61 |
| hco-miR167n; HcARF8; HcTPS8 | miR167n targets the auxin-response factor HcARF8, a negative regulator of floral-fragrance metabolism; HcARF8 binds the promoter of the terpene synthase gene HcTPS8 and represses promoter activity. | *Hedychium coronarium* | Floral fragrance/development; no defined external VOC exposure [class (i)] | Auxin-response transcription-factor layer | hco-miR167n targets HcARF8; HcARF8 binds the HcTPS8 promoter and reduces its activity. STTM/VIGS assays support opposite effects of miR167n and HcARF8 on fragrance metabolism; effects on the overall volatile profile are non-uniform. | Not examined | Floral developmental stages with transient functional assays. | sRNA/degradome, dual-luciferase/Y1H and volatile profiling. STTM-based miRNA interference, BSMV-mediated VIGS, IAA and PCIB treatments. | Functional RNA-level involvement | 62 |
| miR159; miR838 and other candidates; predicted HMGR, camphene synthase, bornyl diphosphate synthase, myrcene synthase; AP2/ERF, WRKY, bHLH, bZIP candidates | Predicted regulation of MVA, mono-, sesqui- and diterpenoid pathways | Lamiaceae species | No defined VOC exposure [class (i)] | Not examined | Predicted post-transcriptional miRNA–target relationships only | Not examined | Computational/transcriptome-mining study; no exposure time course. | Transcriptome mining and computational target prediction; no experimental validation. | Candidate RNA-level association | 63 |
| CsALKBH4A; CsALKBH4B; terpenoid-pathway transcripts | CsALKBH4A/B are m⁶A RNA demethylases that regulate stability/abundance of terpenoid-biosynthesis transcripts. | *Camellia sinensis* cv. Tieguanyin | Solar-withering under graded shading; no airborne VOC exposure [class (v)] | Not resolved | Solar withering altered the m⁶A methylome. CsALKBH4A/B-mediated RNA demethylation affected stability/abundance of terpenoid-pathway transcripts; silencing either eraser increased m⁶A and reduced several volatile terpenoids. | Not examined | 45-min solar-withering under four shading/light-intensity conditions; CsALKBH4A/B silencing experiments analysed separately. | MeRIP-seq/RNA-seq, mRNA-stability assays, silencing of CsALKBH4A/B and GC-MS volatile analysis. | Functional epitranscriptomic involvement | 66 |
| SlYTH2; SlHPL; SlCCD1B | SlYTH2 is an m⁶A reader; SlHPL and SlCCD1B encode enzymes contributing to fatty-acid- and carotenoid-derived aroma volatiles. | *Solanum lycopersicum* cv. Ailsa Craig | Fruit development/ripening; no external VOC exposure [class (i)] | Not examined | SlYTH2 binds m⁶A-modified SlHPL and SlCCD1B transcripts, promotes RNA–protein condensates and represses translation. SlYTH2 knockout increases fatty-acid- and carotenoid-derived aroma volatiles. | Not examined | Tomato fruit-ripening series. | m⁶A-seq/FA-CLIP, polysome/proteomic analyses, CRISPR/Cas9 SlYTH2 knockout and m⁶A-site mutagenesis. | Functional epitranscriptomic involvement | 67 |
| SlYTH1; SlBCAT1; SlTNH1 | SlYTH1 is an m⁶A reader; SlBCAT1 and SlTNH1 encode key enzymes in 2-isobutylthiazole biosynthesis. | *Solanum lycopersicum* cv. Ailsa Craig | Fruit development/ripening; no external VOC exposure [class (i)] | Not examined | SlYTH1 binds m⁶A-modified SlBCAT1 and SlTNH1 and promotes their mRNA stability and translation. Loss of SlYTH1 reduces these targets and lowers 2-isobutylthiazole during ripening. | Not examined | Tomato fruit-ripening series. | m⁶A-target binding, mRNA-stability and translation assays with CRISPR/Cas9 SlYTH1 knockout and GC-MS volatile profiling. | Functional epitranscriptomic involvement | 68 |
| Growth- and defence-related genes | Receiver biomass allocation and defence responses | *Hordeum vulgare* | Naturally emitted constitutive VOC blends from undamaged barley neighbours (emitter–receiver) [class (ii)] | Specific early signalling components not resolved | Epigenetic integration not examined | No priming or stress memory test | Long-term twin-chamber exposure from the seedling stage until harvest. | Twin-chamber emitter–receiver design with RNA-seq and biomass/trait measurements. Demonstrates airborne neighbour effects; no chromatin endpoint or manipulation. | Signalling/transcriptional response only; no chromatin endpoint | 73 |
| LLP1; JAR1; isoprene-associated emitter VOCs | LLP1-associated systemic immune signalling contributes to defence induced by birch VOC blends. | Betula pendula emitters; Arabidopsis thaliana receivers | Field birch–Arabidopsis emitter–receiver exposure; transgenic high-isoprene lines [class (ii)]. | Enhanced resistance requires receiver LLP1; response persists in jar1 mutants. | Not examined (no chromatin- or RNA-level endpoint). | Pathogen resistance after exposure; no cue-removal recovery/challenge design testing stress memory. | Two field seasons; 84 h VOC exposure, then bacterial challenge; bacterial titres after 3 d. | Emitter transgenic birch lines, VOC profiling; receiver wild-type, llp1 and jar1 mutants; Pseudomonas syringae infection assay. | Signalling/defence only; VOC mixture prevents uniquely attributing field response to isoprene; no epigenetic endpoint. | 104 |
| AZI1; EARLI1; AZI3; AZI4; SA/SAR-associated genes | AZI1-family proteins and the SA/SAR network contribute to systemic acquired resistance | *Arabidopsis thaliana* | Controlled headspace exposure to α-pinene + β-pinene; also infected-emitter → receiver exposure [class (iii)] | ROS accumulation; SA signalling; AZI1-dependent resistance | Epigenetic integration not examined | Induced resistance after exposure; chromatin-dependent stress memory not tested | Controlled headspace exposure with receiver-resistance assays. | Headspace exposure with transcriptomics, ROS assays and genetic mutants. Tests defence signalling and SAR; no chromatin or DNA-methylation endpoint. | Signalling only; no chromatin endpoint | 76 |
| MPK3; MPK6; stomatal-immunity components | MAP kinases contribute to (Z)-3-hexenyl butyrate (HB)-induced stomatal immunity | *Solanum lycopersicum* and *Arabidopsis thaliana* | Controlled airborne HB exposure for whole-plant resistance; direct application for mechanism [class (iii)] | Ca²⁺ signalling; NADPH oxidase-derived ROS; MPK3/MPK6 activation; ABA-independent stomatal closure | Epigenetic integration not examined | Enhanced resistance to *Pseudomonas syringae* after airborne exposure; stress memory not tested | Whole-plant airborne HB exposure for 24 h; short direct-application assays for mechanism. | Inhibitor assays, genetics and pathogen assays. EGTA, DPI, SHAM and PD98059, Arabidopsis mpk3/mpk6 and tomato flacca were used to resolve signalling; no chromatin endpoint. | Signalling only; no chromatin endpoint | 77 |
| GLR3.3; GLR3.6; VSP1; acetophenone/octanal/alkanes | GLR-associated emitter volatile production and receiver Ca²⁺/VSP1 signalling after herbivory. | Arabidopsis thaliana (Col-0) | Herbivore-damaged emitter → receiver blend; split-plate assay [class (ii)]. | Receiver Ca²⁺ signal and VSP1 reporter; emitter and receiver glr3.3 glr3.6 requirements. | Not examined (no chromatin or RNA-epigenetic endpoint). | Immediate signalling; no recovery/challenge or chromatin-dependent memory test. | Time-lapse imaging; GC–MS emitter-headspace capture over 3 h; precise imaging durations vary by assay. | Emitter/receiver double mutants, VSP1 fluorescent reporter, GCaMP Ca²⁺ imaging and emitter headspace GC–MS. Individual candidate VOCs not causally tested. | Signalling only; blends and GLR dependence do not identify direct VOC receptors or chromatin mechanisms. | 78 |
| Ca²⁺-permeable channel activity; no single target gene established | Early cytosolic Ca²⁺ signalling in response to plant volatiles | *Arabidopsis thaliana* | Direct exposure to defined plant volatiles including (E)-2-hexenal [class (iii)] | Cytosolic Ca²⁺ transients; ROS-sensitive channel activity | Epigenetic integration not examined | Not examined | Short-term cellular assays (minutes). | Cellular Ca²⁺ imaging and physiological assays; no chromatin endpoint. | Signalling only; no chromatin endpoint | 79 |
| Ca²⁺ signalling components; defence-responsive genes | Sensory Ca²⁺ transduction of green leaf volatiles | *Arabidopsis thaliana* | Controlled green leaf volatile exposure [class (iii)] | Rapid Ca²⁺ signals followed by Ca²⁺-dependent defence-gene activation | Epigenetic integration not examined | Not examined | Short-term Ca²⁺ imaging/expression assays. | Ca²⁺ imaging and functional signalling assays; no chromatin endpoint. | Signalling only; no chromatin endpoint | 80 |
| GCN5 | Histone acetyltransferase and chromatin regulator responsive to cellular redox state | *Arabidopsis thaliana* | H₂O₂/redox treatment; non-VOC redox context [Supporting context] | H₂O₂-associated redox signalling | GCN5 S-sulfenylation at defined cysteine residues provides a direct redox-to-chromatin-modifier interface | Not examined | H₂O₂/redox assays; no VOC exposure. | Nuclear sulfenome/cysteine-redox analyses show redox regulation of GCN5 but do not test a VOC → chromatin pathway. | Supporting context; non-VOC | 81 |
| Bowman–Birk-type trypsin inhibitor gene | Anti-herbivore defence protein | *Zea mays* | HIPVs emitted by herbivore-infested conspecifics (emitter–receiver) [class (ii)] | Upstream early signalling not resolved | Preferential demethylation at selected cytosines in the trypsin-inhibitor promoter accompanies enhanced transcription; the methylation state was not followed through the post-exposure recovery series. | Primed anti-herbivore response remained detectable for up to 5 d after exposure but not at 10 d. Persistence of promoter demethylation was not demonstrated. | Emitter exposure followed by receiver assays across a post-exposure time series; promoter methylation was assayed at a single comparison point. | Bisulfite sequencing, qRT-PCR and larval-performance assays; methylation was not experimentally manipulated. | Association at defined promoter sites | 69 |
| PR1; TI | Defence genes induced in receiver soybean | *Glycine max* | Emitter–receiver exposure to constitutive mint VOC blends; authentic mint volatiles also tested [class (ii)] | Upstream signalling not resolved | Increased histone acetylation at the PR1 and TI promoters; garcinol attenuated the volatile-induced increase in defence-gene transcripts. | In proximate co-cultivation, elevated defence-gene expression and promoter acetylation persisted after mint removal; in a separate greater-distance setup, subsequent herbivore challenge revealed primed defence responses. Histone acetylation was not directly demonstrated as the mechanism of the distance-dependent priming. | Proximate mint co-cultivation for 3 or 7 d with post-removal follow-up; promoter acetylation assayed at 0, 8 and 12 d after source removal; separate distance experiments tested priming. | Emitter–receiver exposure with ChIP, qRT-PCR and defence assays. The HAT inhibitor garcinol weakened the volatile-induced transcriptional response. | Functional involvement (histone acetylation) | 71 |
| ERF8; ERF104; HAC1; HAC2; HAC5; HAM1; HDA6 | ERF8/ERF104 are defence-related transcription factors; HAC1/HAC5/HAM1 contribute to histone acetylation, HAC2 to the defence phenotype, and HDA6 to reversal of the acetylated state | *Arabidopsis thaliana* | Controlled airborne (E)-β-ocimene exposure [class (iii)] | JA/ethylene-associated defence signalling implicated; the upstream receptor is not established | β-Ocimene increased histone acetylation at ERF8/ERF104. hac1, hac5 and ham1 mutants impaired acetylation, while hac1, hac2, hac5 and ham1 mutants impaired the anti-herbivore response; HDA6 contributed to reversal of the acetylated state. | Enhanced anti-herbivore defence persisted for 5 d but not 10 d after exposure. Histone acetylation was functionally linked to the β-ocimene response, but its requirement for persistence was not directly tested. | Six-day headspace β-ocimene exposure in defined developmental windows; defence/chromatin outcomes were assessed immediately or after 5- or 10-d post-exposure intervals, depending on the window. | Defined airborne exposure with chromatin, expression and herbivore assays. Garcinol, HAT mutants and HDA6 analyses tested histone-acetylation machinery. | Functional involvement (histone acetylation) | 70 |
| DDM1; NRPD1; SUVH4/5/6; HDA6; HSFA2 | Regulators of heterochromatin/DNA methylation, RdDM, H3K9 methylation and histone deacetylation tune VOC-induced heat-shock signalling and thermotolerance. | Arabidopsis thaliana Col-0; ddm1-2, nrpd1-3, suvh4/5/6 and hda6 mutants. | Controlled airborne exposure: VOC placed on sterile tissue/filter paper attached to the lid above 7-day-old Arabidopsis seedlings on ½ MS; plate sealed for 30 min [class (iii)]. | Seven-VOC screen induced HSFA2; T2H elicited partly shared heat- and ROS-responsive transcriptional programmes. Direct VOC receptor/early Ca²⁺ signalling not measured. | No chromatin marks were directly assayed after VOC treatment. H3Ac/H3K4me2 promoter differences and HDA6 occupancy were evaluated by reanalysis of independent public WT/hda6 ChIP-seq datasets. | A 30-min VOC pulse followed by 48-h recovery enhanced survival after subsequent lethal heat across genotypes; T2H increased the proportion of healthy hda6 seedlings relative to WT; no causal VOC-induced chromatin memory state demonstrated. | 30-min VOC exposure followed by 48-h recovery, a heat challenge (45 °C, 30 min) and 7-d recovery before survival scoring. VOC doses were selected through preliminary optimisation experiments. | Sealed plate with VOC-loaded sterile tissue/filter paper in lid; empty-paper controls. HSFA2/HSFA1/HSP101 qRT-PCR, survival/phenotype scoring, RNA-seq in Col-0/hda6 and reanalysis of independent public ChIP-seq. No VOC-induced ChIP or epigenome editing. | Functional modulation by chromatin-regulator genotypes; chromatin associations rely on unrelated public ChIP-seq, not matched VOC-exposed samples. Neither a particular VOC-induced chromatin mark nor locus-specific epigenetic causality was established. | 88 |
| EIN3A; CMT2; senescence-associated gene (SAG) promoters | EIN3A represses the DNA methyltransferase CMT2; reduced CMT2-dependent CHH methylation facilitates SAG activation | *Brassica rapa* (non-heading Chinese cabbage) | Controlled gaseous ethylene exposure (canonical hormone-signalling system) [class (iii; gaseous hormone)] | Canonical ethylene signalling via EIN3A | Reduced CMT2-dependent CHH methylation; lower methylation at selected SAG promoters is associated with enhanced EIN3A occupancy and SAG activation. | Senescence progression; no priming or stress memory test | Controlled ethylene treatment for 3 d. | WGBS, ChIP-qPCR, reporter assays, CMT2/EIN3A VIGS and 5-azacytidine treatment. | Functional involvement (DNA-methylation machinery) | 85 |
| HAF2; EIN2-C; ethylene-responsive loci | HAF2 is a histone acetyltransferase recruited to ethylene-responsive chromatin via EIN2-C | *Arabidopsis thaliana* | Controlled gaseous ethylene exposure (canonical hormone-signalling system) [class (iii; gaseous hormone)] | Canonical ethylene signalling/EIN2 | Increased H3K14ac and H3K23ac at ethylene-responsive loci; reduced acetylation and impaired induction in haf2 mutants | No priming or stress memory test | Ethylene gas exposure for molecular analyses (4 h). | Protein-interaction, ChIP-seq/ChIP, mRNA-seq and phenotyping with haf2 mutants, complementation and gain-of-function analyses. Tests canonical ethylene–chromatin coupling, not general plant–plant VOC perception. | Functional involvement (histone acetylation) | 87 |
| DRM2; NRPE1; RDR6; DNA glycosylase genes; PR and other defence genes | Components of the RdDM and active-demethylation pathways together with defence pathways | *Picea abies* | Direct MeJA application [class (iv)] | JA, SA and ethylene pathway transcription induced | Long-term changes in expression of RdDM and active-demethylation components; DNA methylation itself was not measured. | Long-term induced resistance and primed defence-gene expression; not evidence of VOC-specific chromatin memory. | MeJA response followed from hours to 4 weeks; later pathogen challenge. | Time-course transcriptomics and pathogen-resistance bioassays; DNA methylation was not measured. | Indirect association; methylation machinery transcripts only | 83 |
| ROS1; AGO1; RdDM machinery; ATREP2 transposable elements | Demethylation and small-RNA machinery required for long-lasting JA-induced immunity | *Arabidopsis thaliana* | Direct JA application [class (iv)] | JA defence signalling | Selective ATREP2 hypomethylation rather than global hypomethylation; requirement for RdDM, ROS1 and AGO1 | Long-lasting JA-induced resistance remained after a 3-week recovery and was revealed by subsequent challenge; this direct hormone-application design does not test VOC-specific stress memory. | JA treatment followed by a 3-week recovery before long-term-state analysis/challenge. | WGBS, RNA-seq/sRNA, genetics and resistance phenotyping. RdDM mutants, ros1, ago1 and MYC2/3/4 genetics tested requirements for the long-lasting JA response; airborne perception was bypassed. | Functional involvement (DNA-methylation machinery) | 84 |
| OsBBPI; OsPOX | Defence genes responsive to wounding and to MeJA priming | *Oryza sativa* cv. Carnaroli | Direct foliar MeJA followed by mechanical wounding [class (iv)] | JA-dependent priming of the wound response | Increased H3K9ac at selected OsBBPI promoter regions together with changes in global DNA methylation levels. | Primed transcriptional response to the subsequent wounding challenge | MeJA pretreatment; mechanical wounding 24 h later; samples through 48 h post-wounding. | Pretreatment–challenge design with ChIP-qPCR and DNA-methylation assays; chromatin marks were not directly perturbed. | Association of chromatin marks with priming | 82 |
| HDA6; genome-wide stress and specialised-metabolism genes | HDA6 integrates jasmonate-responsive chromatin states | *Arabidopsis thaliana* | Direct exogenous MeJA application to seedlings [class (iv)] | JA-triggered transcriptional programme | Genome-wide H4ac and H3K27me3 remodelling included both MeJA- and HDA6-dependent changes at stress- and specialised-metabolism genes and TEs. | Acute response; priming and stress memory not tested | 50 µM MeJA for 3 h in 12-day-old seedlings. | ChIP-seq/ChIP-qPCR in wild-type and hda6 (axe1-5) plants with or without MeJA. Tests HDA6 involvement in the chromatin response to direct MeJA; airborne perception was not tested. | Functional involvement (histone deacetylation) | 86 |
| No single causal target gene established | Floral signalling and volatile emission under herbivory | *Brassica rapa* | *Pieris brassicae* caterpillar herbivory [class (v)] | Herbivory- and JA-associated defence signalling | Reduced global DNA methylation (MSAP) accompanies altered volatile blends, including lower emission of benzyl nitrile and methyl anthranilate, and reduced flower attractiveness | Methylation and phenotype detectable 2 weeks after treatment; not tested as stress memory | 24-h caterpillar herbivory; methylation/VOCs assessed 2 weeks later. | MSAP, floral VOC profiling and pollinator-behaviour assays. Herbivory alters multiple pathways and does not isolate VOC perception; methylation was not manipulated within the same causal chain. | Association | 46 (herbivory arm) |
| No single causal target gene established | Same system, hormone arm: floral signalling and volatile emission after direct MeJA | *Brassica rapa* | Direct foliar MeJA application [class (iv)] | JA defence signalling | Reduced global DNA methylation (MSAP) with a volatile blend that differs from the herbivory arm; aromatic, terpenoid and nitrogenous volatiles decrease while (Z)-3-hexenyl acetate increases | Detectable 2 weeks after treatment; not tested as stress memory | MeJA sprayed on two consecutive days; methylation/VOCs assessed 2 weeks later. | MSAP and floral VOC profiling. Direct MeJA bypasses airborne perception. 5-azacytidine was tested separately and therefore does not establish a MeJA → methylation → VOC causal chain. | Association | 46 (MeJA arm) |
| miR2977; SAMT | miR2977 post-transcriptionally represses salicylate O-methyltransferase (SAMT), which controls MeSA production and emission | *Citrus reticulata* cv. Shatangju | CLas infection induces MeSA; controlled airborne MeSA; foliar application; SAMT-overexpressing emitter–receiver experiments [class (ii)] | Defence-gene induction downstream of MeSA; detailed early signalling not resolved | Post-transcriptional miRNA control of VOC production in the emitter; receiver experiments measured airborne MeSA signalling, not ncRNA or chromatin changes. | Airborne and exogenous MeSA enhance defence and HLB tolerance; no formal recovery/challenge memory design | Airborne MeSA 1–3 d; complementary foliar and emitter–receiver experiments included longer disease assays. | miRNAome/transcriptome, transgenic lines, GC-MS and disease assays. miR2977 and SAMT were manipulated in the emitter-side pathway; receiver experiments tested airborne MeSA responses, not chromatin regulation. | Functional RNA-level involvement in emitter; receiver signalling without chromatin endpoint | 26 |
| pri-miR163; pri-miR840; MBS1 | Selected primary miRNAs respond to β-cyclocitral headspace exposure and high-light/¹O₂ retrograde signalling; MBS1 contributes to the high-light response. | *Arabidopsis thaliana* | Controlled headspace β-cyclocitral exposure [class (iii)] | β-CC/¹O₂ retrograde signalling; upstream perception not defined | β-CC exposure increased pri-miR163 at both tested loadings and pri-miR840 at the higher loading. Downstream miRNA-mediated target regulation was not tested. | Not examined as priming or stress memory. | 4 h headspace β-CC exposure; MBS1 was analysed separately under high light. | qRT-PCR/small-RNA analyses after β-CC headspace exposure. mbs1 was tested separately under high light; no chromatin endpoint. | RNA-level response; MBS1 support from separate high-light assays | 89 |
| β-Carotene oxidation products; global small-RNA pool | ¹O₂-dependent oxidation of β-carotene generates β-cyclocitral; high light remodels the small-RNA pool and stress/detoxification gene expression | *Arabidopsis thaliana* | High-light stress context; not a β-CC exposure experiment [Supporting context] | ¹O₂/retrograde signalling | High light promotes β-carotene oxidation and β-cyclocitral formation and also alters the small-RNA pool, but the broad small-RNA response cannot be assigned specifically to β-cyclocitral. | Not examined | High-light context studies (hours to days); not β-CC exposure experiments. | Small-RNA sequencing/expression studies; included only as supporting high-light context. | Supporting context; not a β-CC exposure experiment | 30, 90 |
| miR160; miR167; miR172; miR319; miR482/2118 family and predicted targets | Post-transcriptional regulation of NBS-LRR immune receptors and jasmonate-related genes | *Picea abies* | Direct MeJA application followed by a later mechanical wounding challenge [class (iv)] | JA-responsive pathways | Long-lasting changes in miRNA abundance; predicted targets included NBS-LRR immune receptors and jasmonate-related regulators, but miRNA and target expression showed limited overall correspondence; no chromatin endpoint. | miRNA changes persisted for 4 weeks, and a subset showed altered responses to later wounding. The role of individual miRNAs in persistence or priming was not tested. | MeJA treatment; bark challenged by wounding 4 weeks later and sampled 24 h after challenge. | Time-course miRNA profiling with a 4-week challenge interval. Direct MeJA treatment; no miRNA manipulation and no airborne VOC exposure. | RNA-level association with prolonged/challenge-dependent profiles | 91 |
| miR397-z; miR1144-z; miR5658-z; miR5185-z and novel miRNAs | miRNA–mRNA networks associated with heat-stress signalling, proteostasis, cell-wall metabolism and chlorophyll degradation | *Lolium perenne* | Direct MeJA pretreatment followed by heat stress [class (iv)] | JA-associated preconditioning of the heat response | Post-transcriptional miRNA–mRNA network changes; no demonstrated chromatin reprogramming | Improved heat tolerance after pretreatment; stress memory not established | Repeated MeJA pretreatment over 7 d followed by 12 h heat stress. | Integrated miRNA/mRNA sequencing and physiological analyses. MeJA pretreatment altered the heat-stress response, but individual miRNAs were not manipulated. | RNA-level association with altered heat-stress response | 92 |

Note: Evidence categories reflect the experimental endpoints and manipulations reported in each study. Functional involvement of chromatin regulators does not, by itself, establish locus-specific epigenetic causality.

Abbreviations: 5mC, 5-methylcytosine; ATAC-seq, assay for transposase-accessible chromatin sequencing; BSP, bisulfite sequencing PCR; BSMV, barley stripe mosaic virus; ChIP, chromatin immunoprecipitation; CLas, ‘*Candidatus Liberibacter asiaticus*’; CRISPR/Cas9, clustered regularly interspaced short palindromic repeats/CRISPR-associated protein 9; DHS, DNase I hypersensitive site; DMSO, dimethyl sulfoxide; DPI, diphenyleneiodonium; EGTA, ethylene glycol-bis(β-aminoethyl ether)-N,N,N′,N′-tetraacetic acid; EMSA, electrophoretic mobility shift assay; FA-CLIP, formaldehyde cross-linking immunoprecipitation; GC-MS, gas chromatography–mass spectrometry; GNAT, GCN5-related N-acetyltransferase; HAT, histone acetyltransferase; HB, (Z)-3-hexenyl butyrate; HDAC, histone deacetylase; HIPV, herbivore-induced plant volatile; HLB, Huanglongbing; IAA, indole-3-acetic acid; IBT, 2-isobutylthiazole; JA, jasmonic acid; lncRNA, long non-coding RNA; MAPK, mitogen-activated protein kinase; MeJA, methyl jasmonate; MeRIP-seq, methylated RNA immunoprecipitation sequencing; MeSA, methyl salicylate; miRNA, microRNA; mRNA, messenger RNA; MSAP, methylation-sensitive amplified polymorphism; NADPH, nicotinamide adenine dinucleotide phosphate; NBS-LRR, nucleotide-binding site leucine-rich repeat; PCIB, p-chlorophenoxyisobutyric acid; qRT-PCR, quantitative reverse-transcription PCR; RdDM, RNA-directed DNA methylation; RLM-RACE, RNA ligase-mediated rapid amplification of cDNA ends; RNAi, RNA interference; ROS, reactive oxygen species; SA, salicylic acid; SAR, systemic acquired resistance; SBHA, suberoyl bis-hydroxamic acid; SHAM, salicylhydroxamic acid; sRNA, small RNA; STTM, short tandem target mimic; TE, transposable element; TF, transcription factor; TRV, tobacco rattle virus; TSA, trichostatin A; VIGS, virus-induced gene silencing; VOC, volatile organic compound; WGBS, whole-genome bisulfite sequencing; Y1H, yeast one-hybrid.
